# Task-related brain functional network reconfigurations relate to motor recovery in chronic subcortical stroke

**DOI:** 10.1038/s41598-021-87789-5

**Published:** 2021-04-19

**Authors:** Hsiao-Ju Cheng, Kwun Kei Ng, Xing Qian, Fang Ji, Zhong Kang Lu, Wei Peng Teo, Xin Hong, Fatima Ali Nasrallah, Kai Keng Ang, Kai-Hsiang Chuang, Cuntai Guan, Haoyong Yu, Effie Chew, Juan Helen Zhou

**Affiliations:** 1 Center for Sleep and Cognition, Yong Loo Lin School of Medicine, National University of Singapore, Singapore, Singapore; 2 Department of Biomedical Engineering, Faculty of Engineering, National University of Singapore, Singapore, Singapore; 3 Institute for Infocomm Research, Agency for Science Technology and Research, Singapore, Singapore; 4 National Institute of Education, Nanyang Technological University, Singapore, Singapore; 5 Singapore Bioimaging Consortium, Agency for Science Technology and Research, Singapore, Singapore; 6 Queensland Brain Institute and Centre for Advanced Imaging, The University of Queensland, Brisbane, Australia; 7 School of Computer Science and Engineering, Nanyang Technology University, Singapore, Singapore; 8 Center for Translational Magnetic Resonance Research, Yong Loo Lin School of Medicine, National University of Singapore, Tahir Foundation Building (MD1), 12 Science Drive 2, #13-05C, Singapore, 117549 Singapore; 9 Division of Neurology/Rehabilitation Medicine, National University Hospital, Singapore, Singapore; 10 Department of Medicine, Yong Loo Lin School of Medicine, National University of Singapore, 1E Kent Ridge Road, NUHS Tower Block, Level 11, Singapore, 119228 Singapore; 11 Department of Electrical and Computer Engineering, National University of Singapore, Singapore, Singapore; 12 Integrative Sciences and Engineering Programme (ISEP), National University of Singapore, Singapore, Singapore

**Keywords:** Stroke, Neuroscience, Motor control

## Abstract

Stroke leads to both regional brain functional disruptions and network reorganization. However, how brain functional networks reconfigure as task demand increases in stroke patients and whether such reorganization at baseline would facilitate post-stroke motor recovery are largely unknown. To address this gap, brain functional connectivity (FC) were examined at rest and motor tasks in eighteen chronic subcortical stroke patients and eleven age-matched healthy controls. Stroke patients underwent a 2-week intervention using a motor imagery-assisted brain computer interface-based (MI-BCI) training with or without transcranial direct current stimulation (tDCS). Motor recovery was determined by calculating the changes of the upper extremity component of the Fugl–Meyer Assessment (FMA) score between pre- and post-intervention divided by the pre-intervention FMA score. The results suggested that as task demand increased (i.e., from resting to passive unaffected hand gripping and to active affected hand gripping), patients showed greater FC disruptions in cognitive networks including the default and dorsal attention networks. Compared to controls, patients had lower task-related spatial similarity in the somatomotor–subcortical, default–somatomotor, salience/ventral attention–subcortical and subcortical–subcortical connections, suggesting greater inefficiency in motor execution. Importantly, higher baseline network-specific FC strength (e.g., dorsal attention and somatomotor) and more efficient brain network reconfigurations (e.g., somatomotor and subcortical) from rest to active affected hand gripping at baseline were related to better future motor recovery. Our findings underscore the importance of studying functional network reorganization during task-free and task conditions for motor recovery prediction in stroke.

## Introduction

Stroke is caused by interrupted or reduced blood supply to the brain and results in focal lesions^1^. Changes in blood supply after stroke directly impact the physiological parameters of the blood-oxygenation-level-dependent (BOLD) signal measured by functional magnetic resonance imaging (fMRI)^2^, making it a potential tool to investigate brain functional alterations after stroke^2^. The intrinsic functional connectivity (FC) approach maps the spatially distributed, synchronous low-frequency BOLD signal fluctuation and delineates several brain functional networks formed by highly correlated BOLD signals at task-free condition in healthy individuals^3^.

Task-free fMRI has been advocated for investigating brain functional networks in stroke patients due to its high applicability^4,5^. FC between brain hemispheres (inter-hemispheric FC) was associated with motor control^6^, residual motor function^7,8^, and behavioral impairments in attention, memory, language, and vision in stroke patients^7^. Increased FC in ipsilesional dorsal attention and default networks in stroke patients compared to healthy controls was also noted^8^. These findings pointed to network-specific changes at task-free condition after stroke. However, alteration in FC and brain networks corresponding to task demands in stroke patients remains largely unknown. Some studies have found that task-related sensorimotor neural activity was associated with motor recovery in stroke patients^9–11^. Another study using the affected hand gripping task at a target pressure of 10% or 30% of the maximum voluntary contraction for each participant showed FC alterations in brain networks including dorsal attention, visual, motor, and default mode networks and found an positive correlation between ipsilesional sensorimotor activity and motor performance^12^. Previous studies in healthy individuals have demonstrated that FC at rest and task were highly related in terms of activity^13,14^ and topologies^13,15^. Applying fMRI with external task demands may provide more insights in uncovering stroke-induced brain network alterations and its relationship with motor recovery.

While the functional network architecture during rest and task might be highly similar^15,16^, inducing the brain into different states by external cognitive tasks could reveal group differences and individual variations that cannot be readily observed when the brain is in more unconstrainted task-free states^17^. A study in healthy subjects found that higher efficiency in task-related reconfiguration, i.e., higher similarity between rest and task FC patterns, was related to better performance in language, reasoning, and working memory^16^. Network-specific high flexibility changing from rest to task was reported in default, control, and salience networks^18^ and the working memory task-related reconfiguration patterns further revealed the differential roles of the control and default networks in healthy subjects^19^. Nevertheless, little is known about how brain functional networks reconfigure from rest to task in stroke patients and how these functional network disruptions or reorganization at baseline influence post-stroke motor recovery.

Although previous studies have investigated task-free and task-based FC related to motor recovery in stroke patients, there is a lack of studies examining task-free and task-based FC simultaneously in the same cohort of stroke patients. Furthermore, the similarity between task-free and task-based FC architecture remains largely unknown. The influence of external task demands on both FC and brain functional network reconfiguration in stroke patients is less studied. Importantly, how the changes of FC/brain functional network reconfiguration with different task demands associate with motor recovery in stroke patient? To cover these gaps, we adopted a whole-brain network-based approach as previous findings reported various brain networks associated with post-stroke motor function/recovery such as the default, dorsal attention, and somatomotor networks. We examined the differences in both task-free and task-based FC between chronic subcortical stroke patients and age-matched healthy controls. We hypothesized that stroke patients would have lower FC in somatomotor and subcortical networks than healthy controls at both task-free and task-based conditions; more importantly, we expected more FC disruptions, particularly in the high-order cognitive networks, in stroke patients during more demanding task conditions (active movement) compared to passive/assisted movement. We also predicted that stroke patients would have higher task-based reconfigurations in somatomotor and subcortical networks compared to healthy controls. We sought to test if these network-specific brain FC disruptions and task-related reconfigurations at baseline will relate to motor recovery in stroke patients after undergoing a 2-week motor imagery-assisted brain computer interface-based (MI-BCI) training with or without transcranial direct current stimulation (tDCS).

## Results

### More widespread network disruptions during task than during rest condition post-stroke

**Figure 1:**
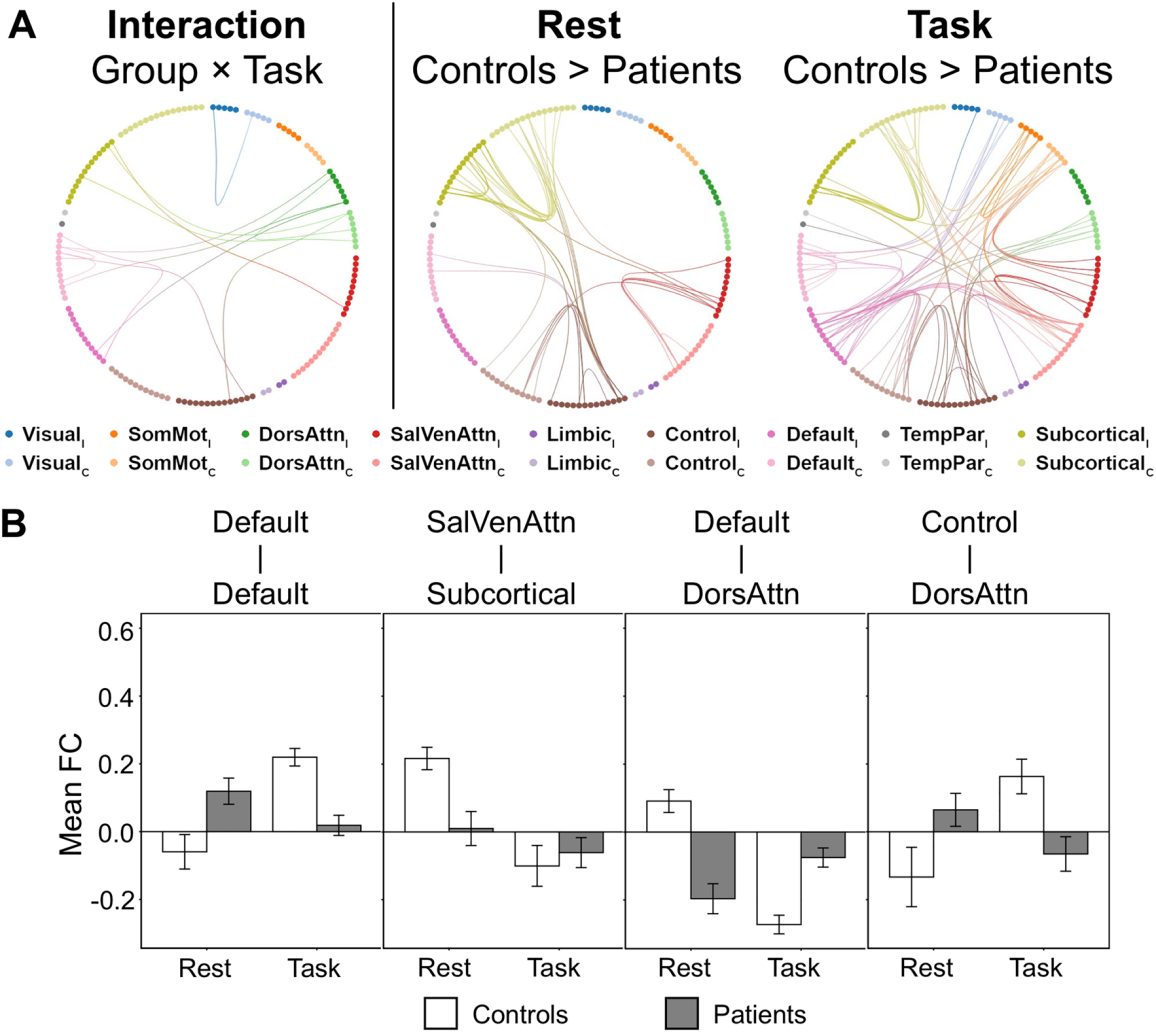
More widespread network disruptions in task-based condition than task-free condition in stroke patients. (**A**) (Left panel) Significant interaction effects of group (patients vs controls) and task (rest vs task) were found in intra-network FC within default and DorsAttn networks and in inter-network FC between default and DorsAttn, control and DorsAttn, SalVenAttn and subcortical networks. (Right panel) Compared to age-matched healthy controls, stroke patients showed lower intra-network FC in control, SalVenAttn, and subcortical networks as well as lower inter-network FC between ipsilesional control and bilateral subcortical networks during the task-free condition. During the task-general condition, stroke patients had more widespread network disruptions than resting-state, including lower intra-network FC in default, control, SalVenAttn, SomMot, and subcortical networks. Additionally, lower inter-network FC was found between ipsilesional default and other contralesional networks (except limbic and subcortical), ipsilesional control and other contralesional networks (except limbic, visual, and TempPar), as well as contralesional SalVenAttn and bilateral SomMot and subcortical networks. (**B**) The average FC of edges showing significant interaction effect for four representative networks impaired based on NBS statistics in panel (**A**) were shown. Data are presented as mean ± standard error. *C* contralesional, *DorsAttn* dorsal attention, *FC* functional connectivity, *I* ipsilesional, *NBS* network-based statistics, *SalVenAttn* salience/ventral attention, *SomMot* somatomotor, *TempPar* temporoparietal.

To investigate the group (stroke patients vs age-matched healthy controls) and task [task-free vs average of four task conditions (i.e., task-general)] interaction, we performed two-way repeated measure analysis of covariance with mean absolute motion displacement as a nuisance covariate using network-based statistic (NBS) toolbox (see “Methods”). Significant group and task interaction effects were observed in default, control, salience/ventral attention, dorsal attention, and subcortical networks (FDR corrected *p* < 0.05, partial η^2^ = 0.024, Fig. 1A,B).

To compare group differences in FC strength at task-free and task-general conditions, we performed two-sample *t* tests using NBS (controlled for mean absolute motion displacement). In the task-free condition, compared to age-matched healthy controls, we observed that stroke patients had lower intra-network FC (NBS corrected *p* = 0.037, Cohen’s d = 0.016) in bilateral control, salience/ventral attention, and subcortical networks as well as lower inter-network FC between ipsilesional control and bilateral subcortical networks (Fig. 1A and Supplementary Figure 2).

In the task-general condition, we observed that stroke patients had more extensive network disruptions in the task condition compared to the rest condition, namely lower intra-network FC in bilateral default, control, salience/ventral attention, somatomotor and subcortical networks. Lower inter-network FC was also found between ipsilesional default and other contralesional networks (except limbic and subcortical), between ipsilesional control and contralesional salience/ventral attention, dorsal attention, somatomotor, and subcortical networks, as well as contralesional salience/ventral attention and bilateral somatomotor and subcortical networks (NBS corrected *p* = 0.007, Cohen’s d = 0.017, Fig. 1A and Supplementary Figure 2).

After performing control analyses (see “Methods”), similar findings were obtained for both scan length (time) matching (i.e., control analysis I) and number of volumes matching analyses (i.e., control analysis II; Supplementary Figure 3) as well as based on the resting and task fMRI data without global signal regression (control analysis III; Supplementary Figure 4). Controlled for handedness and the affected hand (i.e., control analysis IV), we found similar group and task interaction effect at a slightly lower threshold (height uncorrected *p* < 0.01). FC disruption patterns at rest and task (Supplementary Figure 5A) as well as anti-correlated FC patterns in task (Supplementary Figure 5C, left panel) in stroke also remained.

### More disrupted functional connectivity with higher task demands in stroke patients

**Figure 2:**
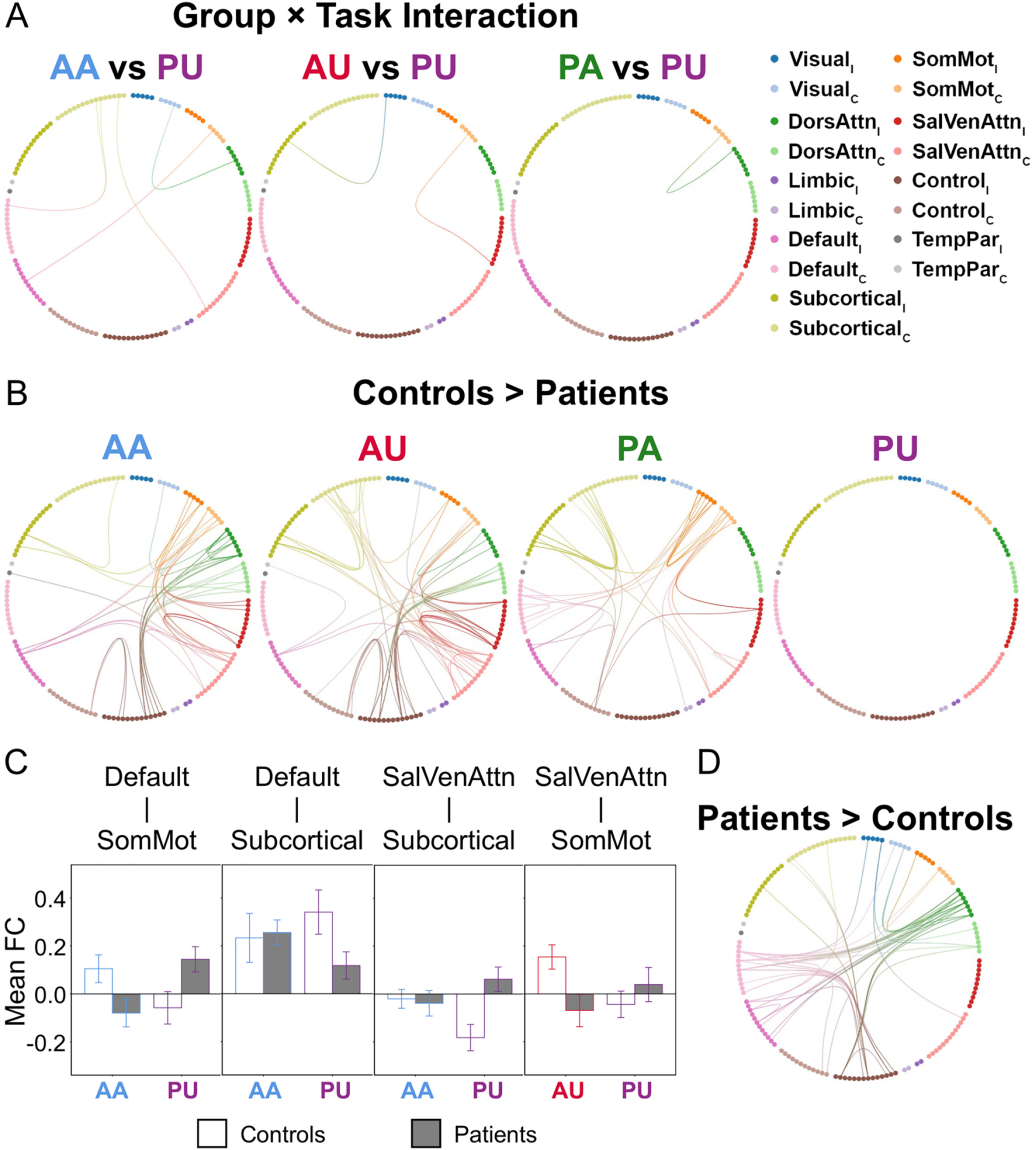
Disrupted intra- and inter-network functional connectivity at different task demands in stroke patients. (**A**) Significant group and task interaction effects were found between AA/AU/PA and PU. (**B**) Compared to age-matched healthy controls, stroke patients showed more disrupted intra- and inter-network FC during AA, AU, and PA tasks. During AA, stroke patients demonstrated lower FC in ipsilesional default as well as bilateral control, SalVenAttn, DorsAttn, SomMot, and subcortical networks. Remarkably, stroke patients presented additionally lower DorsAttn intra-network FC in AA compared to other tasks. Along with the decremental task demands, stroke patients showed less aberrant intra- and inter-network FC disruption. (**C**) The average FC of edges showing significant interaction effects for four representative networks impaired in stroke patients based on NBS statistics in panel (**A**) were shown. (**D**) Especially for the AA task, stroke patients showed more anti-correlation than healthy controls in default, control, and DorsAttn networks. Data are presented as mean ± standard error, *AA* active-affected, *AU* active-unaffected, *C *contralesional, *DorsAttn* dorsal attention, *FC* functional connectivity, *I* ipsilesional, *NBS* network-based statistics, *PA* passive-affected, *PU* passive-unaffected, *SalVenAttn* salience/ventral attention, *SomMot* somatomotor, *TempPar* temporoparietal.

For stroke patients, using the affected hand is naturally more difficult than using the unaffected hand. Given the nature of passive and active tasks, it is also reasonable to assume that active condition is more difficult than passive condition. Consequently, the task using the affected hand actively (i.e., AA) was considered the most difficult one, followed by the task using the unaffected actively (i.e., AU) and the task using the affected hand passively (i.e., PA), and the easiest task was the task using the unaffected hand passively (i.e., PU). Using PU as a reference, we performed three separate two-way repeated measures analysis of covariance (ANCOVA) to examine the effect of group and task (AA/AU/PA versus PU) as well as their interactions using NBS with *p* < 0.05 FDR corrected (see “Methods”). We noted significant group and task interaction in comparisons between AA/AU/PA and PU (all FDR corrected *p* < 0.05, AA vs PU partial η^2^ = 0.093, AU vs PU partial η^2^ = 0.188, PA vs PU partial η^2^ = 0.001, Fig. 2A,C).

In terms of group comparison examined by two-sample t-tests using NBS (controlled for mean absolute motion displacement), we found significant differences between patients and controls in all but the PU task (AA: *p* = 0.028, Cohen’s d = 0.009; AU: *p* = 0.024, Cohen’s d = 0.009; PA: *p* = 0.020, Cohen’s d = 0.021; Fig. 2B and Supplementary Figure 6). Stroke patients showed more network disruptions when performing a task with higher task demands compared to healthy controls.

Specifically, during the AA task, stroke patients presented lower FC than healthy controls (*p* = 0.035, Cohen’s d = 0.009) in ipsilesional default network as well as bilateral control, salience/ventral attention, dorsal attention, somatomotor, and subcortical networks. Compared to AU, PA, and PU, stroke patients showed additionally lower intra-network FC in the dorsal attention network compared to controls.

During the affected-hand tasks (i.e., AA and PA), stroke patients had lower intra-somatomotor network FC and lower inter-network FC between the default and somatomotor relative to healthy controls. This pattern was missing during the unaffected-hand tasks (i.e., AU and PU). Notably, during the AA task, stroke patients demonstrated more anti-correlation in default, control, and dorsal attention networks compared to healthy controls (Fig. 2D and Supplementary Figure 6). This finding implied that the task demand greatly influenced the brain network organization in stroke patients.

Again, the findings of control analysis III demonstrated task-specific network disruption patterns (Supplementary Figure 7) which is similar to our main findings. The findings of control analysis IV showed a significant group and task interaction effect in comparisons between AA/AU/PA and PU (height threshold uncorrected *p* < 0.01), which is similar to what we found in the main analysis (Supplementary Figure 5B, top panel). In terms of group comparisons, significant group differences in AA and AU tasks were also found (see Supplementary Figure 5B, lower panel). Anti-correlated FC patterns in AA were observed as well (see Supplementary Figure 5C, right panel).

### Inefficient task-related network-specific reconfiguration in stroke patients

**Figure 3:**
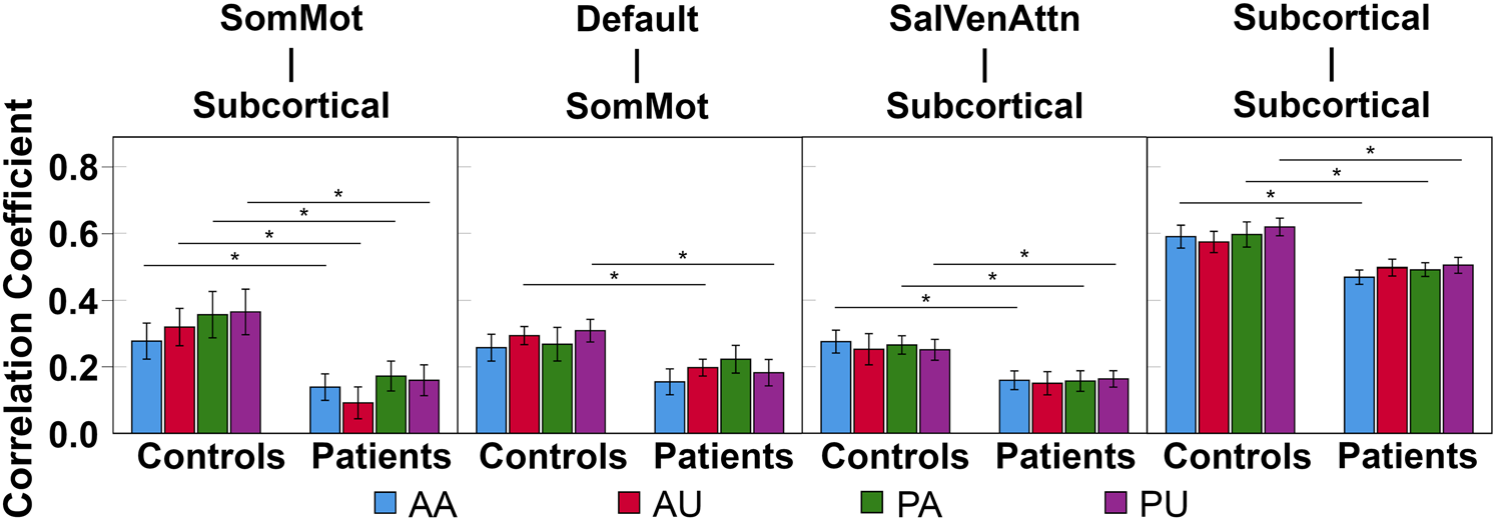
Altered network-specific reconfigurations at different task demands in stroke patients. At the network-level, stroke patients presented network-specific brain network reconfiguration compared to healthy controls. Stroke patients had higher task-related reconfiguration (i.e., lower correlation coefficient) between SomMot and subcortical networks across all tasks. Stroke patients also showed higher reconfiguration between default and SomMot networks during AU and PU tasks. Except the AU task, stroke patients presented higher reconfiguration in SalVenAttn and subcortical inter-network as well as subcortical intra-network. Data are presented as mean ± standard error, *p < 0.05. *AA* active-affected, *AU *active-unaffected, *PA *passive-affected, *PU *passive-unaffected, *SalVenAttn *salience/ventral attention, *SomMot* somatomotor.

Two-sample *t* tests were used to compare the group difference in task-related reconfigurations for every task (see “Methods”). We did not find significant group difference in task-related whole-brain network reconfiguration, although stroke patients showed numerically higher reconfiguration (i.e., lower correlation coefficient) between task-free and task-based condition than healthy controls. In contrast, stroke patients demonstrated more network-specific reconfiguration at different task demands in comparison with healthy controls (Fig. 3). Specifically, stroke patients showed higher reconfiguration in default and somatomotor inter-network during AU and PU. For AA, PA, and PU, stroke patients had higher reconfiguration in salience/ventral attention and subcortical inter-network as well as subcortical intra-network. This might suggest that subcortical stroke has an impact on not only subcortical network but also other networks such as default, somatomotor, and salience/ventral attention. Comprehensive results of reconfiguration are tabulated in Supplementary Table 4.

Control analysis III showed similar findings in somatomotor, salience, and subcortical networks, except that there was no group difference in reconfigurations between the default and somatomotor networks across four tasks (Supplementary Figure 8). Control analysis IV demonstrated task-specific brain network reconfiguration between somatomotor and subcortical networks and within subcortical networks (Supplementary Figure 9).

### Baseline network-specific functional connectivity and task-related brain network reconfiguration were associated with motor recovery in stroke patients

**Figure 4:**
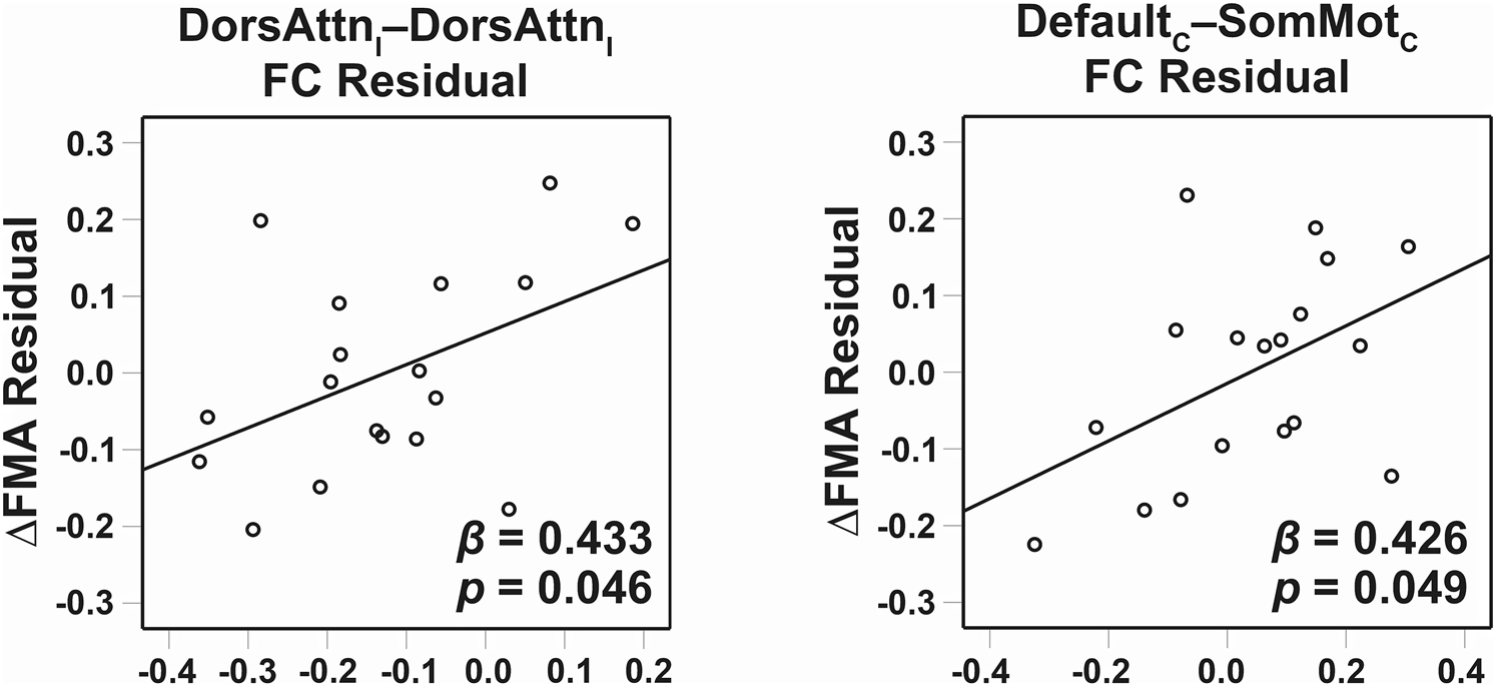
Network-specific functional connectivity of the active-affected task was associated with motor recovery in stroke patients. More FC within ipsilesional DorsAttn network was associated with better motor recovery. More FC between contralesional default and contralesional SomMot was also associated with better motor recovery. *C *contralesional, *DorsAttn *dorsal attention, *FC *functional connectivity, *FMA *Fugl–Meyer Assessment, *I *ipsilesional, *SomMot *somatomotor.

To investigate the association between baseline FC and motor recovery in chronic subcortical stroke patients, we built one stepwise multiple regression model for each task condition by including FC strengths showing group differences as predictors (Fig. 2B; see “Methods”). Out of all conditions tested, two models examining the association of baseline FC at AA and AU conditions with motor recovery (i.e., normalized FMA change score) were significant. The stepwise multiple regression model of AA explained 41.1% of variance (*p* = 0.019) with the involvement of ipsilesional dorsal attention intra-network FC (*β* = 0.433, *p* = 0.046), and contralesional default–somatomotor inter-network FC (*β* = 0.426, *p* = 0.049) (Fig. 4). The stepwise multiple regression model of AU explained 32.1% of variance (*p* = 0.014) with the involvement of ipsilesional dorsal attention intra-network FC (*β* = 0.566, *p* = 0.014). Higher FC at these connections, especially within ipsilesional dorsal attention network, was associated with better motor recovery in chronic subcortical stroke patients.

Similarly, we built one stepwise multiple regression model for each task condition by including brain network reconfiguration showing group differences as predictors (Fig. 3) to investigate the association between baseline reconfiguration measures and motor recovery in stroke patients (see “Methods”). Regarding brain network reconfiguration, we found that only the model using brain network reconfiguration at the AA condition at baseline was found significantly predicting motor recovery. It explained 29.3% of variance (*p* = 0.020) with the involvement of connection reconfiguration between ipsilesional somatomotor network to contralesional subcortical network (*β* = 0.541, *p* = 0.020) (Supplementary Figure 10). This suggested that higher brain network spatial similarity (i.e., lower network reconfiguration from rest to AA) at baseline was associated with more motor gains after intervention in chronic subcortical stroke patients.

## Discussion

To study how brain functional networks reorganize with motor tasks and how these changes at baseline are associated with motor recovery in chronic subcortical stroke, we compared FC strength and network reconfiguration between stroke patients and healthy controls at both task-free and task-based conditions (with different task demands). We found that stroke patients showed more widespread network disruptions during motor task (relative to rest condition) particularly in subcortical, somatomotor, default, and dorsal attention networks compared to healthy controls. We also found more task-specific brain network reconfiguration especially in subcortical and somatomotor networks in stroke patients. Our findings suggested that stroke-induced focal subcortical lesion introduced both local and remote brain functional connectivity changes as well as task-related functional reconfigurations in a network-specific manner, and the brain functional networks involved such as default, attention, somatomotor, and subcortical networks were dependent on the task demands. Importantly, both task-specific FC and brain network reconfiguration during the task using the affected hand actively were associated with motor recovery in chronic subcortical stroke patients. Taken together, network-specific brain network reorganizations at rest and task with varying degree of task demands have the potential to predict motor recovery after rehabilitation in chronic subcortical stroke patients.

Brain functional architecture in healthy individuals are organized into highly specialized networks associated with various functions. FC can reveal specialization (i.e., high correlations within networks), segregation (i.e., low correlations in BOLD signals between networks), and integration (i.e., high correlations between networks)^20^. In terms of group differences, we found lower task-free and task-based FC in stroke patients compared to healthy controls in subcortical, somatomotor, default, control, salience/ventral attention and dorsal attention networks, which was consistent with previous findings^7,12,21^. Furthermore, stroke patients had more widespread FC disruptions during task compared to rest conditions, especially in higher-order cognitive (the default, dorsal attention, control and salience/ventral attention) and subcortical networks. Such phenomenon is even more pronounced during the high demanding tasks (e.g., active affected hand task) in the higher-order cognitive, subcortical, and somatomotor networks. Overall, our findings suggested stroke patients had a loss of network specialization (e.g., intra-network FC disruption in the default network during task), degraded network segregation (e.g., increased FC between salience/ventral attention during task), and less network integration (e.g., decreased FC between control and dorsal attention during task). Our findings are robust as all validation analyses including unequal scan length between rest and task fMRI data, no global signal regression, and the affected hand showed similar findings.

Focal subcortical lesions might influence local and remotely associated cortical regions through extensive white matter tracts and/or damage of the core integrative and associative input to other cortical regions residing at the lesion sites^22^. Stroke-induced reduction of metabolic diaschisis and disruption of inter-regional BOLD signal incoherence are also associated with abnormalities in multiple regions and networks^22^. Indeed, subcortical stroke impairs the integrity of corticospinal system and then influences the recruitment of bilateral secondary motor networks^23^. Additionally, subcortical regions play key roles in the functioning of the salience network and control network^24–26^. Consequently, subcortical stroke leads to widespread network disruptions observed in task-based fMRI^12^.

Dorsal attention network is associated with cognitive function regarding attentional selection and detection of behavioral relevant stimuli after stroke^27^. Altered FC between default, control, and attention networks after subcortical stroke was also reported, suggesting the reorganization of the attention system^28^. In order to perform the most difficult motor task (i.e., AA) at a satisfactory level, patients may have required more attention and action selection resources. Hence, they had to engage not only the subcortical and somatomotor network but also default, control, salience/ventral attention, dorsal attention networks to better execute the task with a planned motor control strategy. Such task-induced increase in brain FC may not be always readily observed using task-free MRI, supporting the complementary value of studying task-based fMRI FC^17^.

Interestingly, task-related reconfiguration patterns demonstrated similar network-specific disruptions observed in task-related FC. During motor task execution, the subcortical network transmits integrated sensorimotor information for motor planning to somatomotor network^29^, and therefore somatomotor–subcortical pathway was the most damaged across tasks. Brain lesions in subcortical regions also impacted brain network reconfiguration within subcortical network and cognitive networks such as salience/ventral attention network, consequently leading to reconfiguration inefficiency across tasks except the AU task. Of the supportive role of the default network in motor function^12^, the default–somatomotor pathway did not show inefficient brain network reconfiguration in affected-hand tasks in chronic subcortical stroke patients. This may imply the adaptive reorganization of the functional networks after stroke^30^. In line with previous findings, network-specific task-related reconfiguration patterns may underline the essential roles of subcortical, somatomotor, and cognitive networks in motor control.

Importantly, less FC disruptions and more efficient task-related reconfiguration at the most demanding task (AA) at baseline predicted motor recovery better than resting state and other conditions with lower demands in chronic subcortical stroke patients. This underscores the importance of evaluating neural networks underpinning using the affected hand actively (AA task). Previous studies suggested that reduced FC in the dorsal attention network may imply limited attention load in stroke patients for dual task demands^31,32^. Moreover, the corticospinal system is activity- and use-dependent^33^. The AA task required high level of attention to visual stimuli and movement and integration of sensory and motor information, many of which subserved by the dorsal attention network^34^. It hence suggested that more preserved FC within ipsilesional dorsal attention plays a facilitatory role in motor recovery.

We noted a difference in FC disruptions between affected-hand tasks (AA and PA), implying differential reorganization pattern mediated by task. The contribution of the contralesional hemisphere to affected-hand function may be resulted from several possible mechanisms of compensation of motor deficits^35,36^. For instance, diminished inhibition from ipsilesional primary motor cortex to contralesional primary motor cortex is a possible mechanism of motor recovery in stroke^35,36^. More activation of the contralesional cortical network was related to better motor recovery^37^ with additional neural resources recruited^38^. As stroke causes widespread brain functional network disruptions with varying degrees, involvement of the contralesional somatomotor network may represent adaptive motor learning strategies^39^. Moreover, newly formed transcallosal connections projecting from the contralesional hemisphere to the ipsilesional hemisphere may enable the contralesional hemisphere to control movements of the affected side^40^. Indeed, our results of diffusion tensor imaging in these patients showed increased fractional anisotropy in the corpus callosum^41^. Involvement of contralesional default network also support residual motor function with extra information integration within its main hubs^12^. Thus, contralesional hemisphere could be potentially involved as a compensatory mechanism for motor recovery in chronic subcortical stroke patients.

In addition, we observed that brain network reconfiguration between ipsilesional somatomotor and contralesional subcortical networks of the AA task predicted motor recovery in chronic subcortical stroke patients. Previous studies suggested that residual corticospinal and cortico-subcortical connections may facilitate motor recovery of the affected hand^42^. More efficient brain network reconfiguration between ipsilesional cortical somatomotor and contralesional subcortical regions via cortico-subcortical connections might hence promote motor recovery. This again supported that contralesional hemisphere may assist ipsilesional hemisphere in integration of somatosensory input and motor control in chronic subcortical stroke patients, indicating that contralesional hemisphere plays an essential role in motor recovery.

A few limitations should be noted. First, our sample size is small and caution needs to be taken when interpreting the current results, which is a pilot study of stroke rehabilitation involving multimodal complex design of neuroimaging, tDCS and MI-BCI. Based on our findings here, we provided effect size and power estimates to associate task-specific FC with motor recovery, which will help design of future task-free and task-fMRI studies with larger sample in stroke rehabilitation (see Supplementary Results). Second, the relative short duration and unequal length between rest and task fMRI data may potentially confound the FC reorganization findings, but a 5 min scan can generate moderate reliability^43^. Additional validation analyses to match the scan time and number of volumes between rest and task showed highly similar findings. Given that individual differences are preserved in FC on top of task modulations^44^, our findings on the rest and task FC changes in stroke patients were less likely to be influenced by short or unequal scan length in terms of time or number of volumes. Third, due to the limited sample size, we were not able to control for stroke severity or lesion size and location. However, we have provided spatial maps of lesion distributions in a network-specific manner (Supplementary Figure 1 and Supplementary Table 3) for interpretations. Future research should incorporate these potential important factors to comprehensively understand brain network reorganization after stroke and their relationship with motor recovery. For example, investigations on the degree of overlap between the lesion mask and the connectivity findings and derivation of seed-based functional connectivity using the cavity as a seed to predict the patterns of brain network reorganization would be worth exploring. Finally, the generalizability of our findings may be limited as only chronic subcortical stroke patients were examined in this study. Future work is needed to further examine and corroborate our findings in stroke patients with different characteristics.

## Conclusions

In conclusion, compared to age-matched healthy controls, chronic subcortical stroke patients had more widespread brain functional network disruptions at task condition relative to rest condition, especially in cognitive, somatomotor, and subcortical networks. Chronic subcortical stroke patients also had less efficient task-related brain network reconfiguration than healthy controls at different task demands. Importantly, baseline network-specific FC and reconfiguration could predict motor recovery. Our findings highlight the necessity of simultaneously examining large-scale brain functional networks at task-free and task-based conditions in stroke patients. Future studies are needed to examine the intervention effect on brain structural and functional network reorganization in association with motor recovery in stroke patients.

## Methods

### Participants

Nineteen chronic subcortical stroke patients and eleven age-matched healthy subjects were recruited in this study. The inclusion criteria for stroke patients were (1) first ever subcortical stroke > 9 months before the study enrolment; and (2) unilateral moderate to severe upper extremity motor impairment measured by the upper extremity component of the Fugl–Meyer Assessment (FMA) (score range 11–45). The exclusion criteria for stroke patients were (1) epilepsy, neglect, cognitive impairment, and other neurological or psychiatric diseases; (2) severe arm pain; (3) severe spasticity measured by the Modified Ashworth Scale in the shoulder or elbow (score > 2); (4) contraindications to tDCS; (5) weak grip strength measured by a dynamometer (weight < 10 kg); and (6) participation in other interventions or trials that target post-stroke motor recovery. All participants performed one baseline MRI scan. All stroke patients underwent an MI-BCI training with or without tDCS for 2 weeks. The combination of MI-BCI and tDCS may promote brain reorganization and functional recovery in stroke patients. Within each session, 20 min of real or sham tDCS were administered followed by 40 min of MI-BCI training. The tDCS was applied at 1 mA with the anode and cathode placed over the ipsilesional and contralesional primary cortex, respectively. A motor-imagined reaching task was used to trigger the InMotion MIT-Manus robot (Interactive Motion Technologies, MA, USA) for upper limb rehabilitation. Stroke patients had their motor function of the affected arm evaluated by the FMA before and two weeks after the intervention (for details see Supplementary Methods^41,45,46^). Here, we refer to the before intervention timepoint as the baseline timepoint. The FMA change scores between baseline and two weeks after intervention did not have significant group difference (mean 5.00 (SD 4.40) in MI-BCI + real tDCS group (n = 10) and mean 5.75 (SD 5.99) in MI-BCI + sham tDCS group (n = 9), *p* = 0.763). One patient was excluded because of excessive motion during the scan. The demographic and clinical information is tabulated in Table 1. More detailed demographic and clinical information in stroke patients is tabulated in Supplementary Table 1.

**Table 1:** Subject demographic and behavioral characteristics.

	Healthy controls (n = 11)	Stroke patients (n = 18)	*p*-value (2-tailed)
Age (years), mean (SD)	56.73 (4.47)	52.89 (10.32)	0.180
Sex (male/female)	6/5	14/4	0.425
Handedness (left/right)	0/11	2/16	0.520
Time post-stroke (months), mean (SD)	–	33.61 (19.80)	–
Affected hemisphere (left/right)	–	8/10	–
Baseline Fugl–Meyer Assessment score, mean (SD)	–	34.33 (7.50)	–
Fugl–Meyer Assessment change score, mean (SD)	–	5.33 (5.02)	–
Baseline grip strength (kg), mean (SD)	–	6.12 (3.94)	–
Baseline box and block test, mean (SD)	–	3.67 (4.50)	–

Independent *t* test was performed to compare the group difference in age. Fisher’s exact test was performed to compare the group difference in sex and handedness separately. There was no significant group difference in age, sex, and handedness (*p* > 0.05). Fugl–Meyer Assessment change score refers to the change after motor imagery-assisted brain computer interface-based training with and without transcranial direct current stimulation intervention (i.e., post-intervention minus pre-intervention).

The stroke lesion was manually drawn by a research fellow (X.H.) based on T_2_-weighted fluid-attenuated inversion recovery images and non-linearly registered to Montreal Neurological Institute (MNI) 152 space. The stroke lesion overlapping map is illustrated in Supplementary Figure 1. This study was conducted in accordance with the Code of Ethics of the World Medical Association and approved by the Domain-Specific Review Board of the National Healthcare Group (NHG) in Singapore. NHG oversees National University Health System including National University Hospital and Yong Loo Lin School of Medicine, National University of Singapore. This study was also registered on ClinicalTrials.gov (Clinical Trial Registration Unique Identifier: NCT01897025 (11/07/2013); https://clinicaltrials.gov/ct2/show/NCT01897025). Written informed consent was obtained from all participants.

### Task-free and motor task-based fMRI

The MRI session included one run of task-free resting state fMRI (7 min) followed by four runs of a block-design hand-gripping task (4.45 min per run). In the task-free run, participants were instructed to close their eyes but remain awake without thinking of anything in particular. In each motor task run, participants were cued to grip a pressure sensor using either their right or left hand actively or passively. The order of the four runs was counter-balanced across participants. Each block began with a cue signaling rest for 33 s, followed by a cue signaling hand gripping/rest for 21 s repeated nine times, and finally ended by another cued rest for 45 s. In each motor task run, participants were cued to grip a pressure sensor at a target pressure of 30% of their maximum voluntary contraction using either their right or left hand actively or passively. The gripping pressure was fixed for four motor tasks and was recorded to evaluate the task performance. The order of the four runs was counter-balanced across participants. A research assistant assisted participants in the passive hand gripping task according to the visual cue whereas participants performed active hand gripping task without assistance. Two tasks required participants to perform hand gripping actively without external assistance were categorized into active task, and two tasks assisted by a research assistant were categorized into passive task. By visual inspecting of the task performance, the peak of each movement onset with attenuation of hand gripping pressure could be clearly identified, indicating that all stroke patients and healthy controls were able to perform the active tasks using either affected or unaffected hand. We additionally investigated whether patients and controls showed any differences in terms of achieving the target pressure per visual cue in the active motor tasks by dividing the number of trials meeting the target pressure by the total number of trials per participant. After performing two-sample t-tests, we did not find any group differences between healthy controls and stroke patients in both active tasks using affected or unaffected hand. For patients with stroke lesions in the right hemisphere, we flipped their MR images (lesion mask, task-free and task-based fMRI) to ensure that lesions were on the left side of the hemisphere. Thereafter, the left hemisphere was regarded as the ipsilesional hemisphere and right hemisphere was regarded as the contralesional hemisphere in stroke patients. For healthy controls, their MR images were not flipped. Accordingly, the right hand was treated as the affected hand and the left hand as the unaffected hand. This resulted in four motor task conditions ordered in decreasing demands: active-affected (AA), active-unaffected (AU), passive-affected (PA), and passive-unaffected (PU).

### Image acquisition

Participants were scanned in a 3 T Siemens Magnetom Trim Trio scanner with a 32-channel head array coil at the Clinical Imaging Research Center, Singapore^41^. The scan protocol included a T_1_-weighted magnetization prepared rapid gradient-echo (MPRAGE) sequence (inversion time = 900 ms, repetition time = 1900 ms, echo time = 2.5 ms, flip angle = 9**°**, field of view = 256 × 256 mm^2^, 176 sagittal slices, matrix size = 256 × 256, and voxel size = 1 mm^3^ isotropic), a task-free T_2_*-weighted echo planar imaging (repetition time = 1750 ms, echo time = 30 ms, flip angle = 74°, field of view = 220 × 220 mm^2^, voxel size = 3.4 mm^3^ isotropic, slice thickness = 3.4 mm, 33 axial slices), and four task-based T_2_*-weighted echo planar imaging (repetition time = 3000 ms, echo time = 30 ms, flip angle = 90°, field of view = 220 × 220 mm^2^, voxel size = 3.4 mm^3^ isotropic, slice thickness = 3.4 mm, 42 axial slices).

### Image preprocessing

Both task-free and task-based fMRI images were preprocessed using the Analysis of Functional NeuroImages software (AFNI)^47^ and FMRIB (Oxford Centre for Functional MRI of the Brain) Software Library (FSL)^48^, The preprocessing steps for task-free data followed our previous protocol^49–51^, consisting of: (i) removal of first six volumes for magnetic field stabilization; (ii) slice-time correction; (iii) motion correction, (iv) despiking; (v) spatial smoothing (6-mm FWHM Gaussian kernel); (vi) grand mean scaling; (vii) band-pass temporal filtering (0.009–0.1 Hz); (viii) detrending (removal of first and second order); (ix) use of Boundary-Based Registration (BBR) for co-registration of T_1_ image^52^ and nonlinear registration tool (FNIRT) for subsequent registration to the MNI 152 space, and (x) regression of nuisance signals (white matter, cerebrospinal fluid (CSF), whole-brain global signals, and six motion parameters).

For task-based data, we performed preprocessing steps (i) (with first five volumes removed), (ii), (iii), (v), and (ix) above. We did not perform despiking, grant mean scaling and band-pass filtering and detrending to avoid removing task-relevant signals following previous literature^15,49–51,53^. Furthermore, we used the Statistical Parametric Mapping toolbox (SPM12) (http://www.fil.ion.ucl.ac.uk/spm/) to regress out the global signal, white matter, CSF, and motion parameters as well as the task-related mean activation signals^15,53^ by modeling the onset of each task block as a boxcar regressor convolved with a generic hemodynamic response function in the same general linear model in (x). Subsequently, the residual task-free and task-based signals were used for FC analysis. Voxels within stroke lesion masks were not excluded during preprocessing but the visual inspection results did not reveal influences of stroke lesions.

To ensure adequate confounds control, we visually inspected the quality of co-registration and normalization. Motion scrubbing was performed on task-free fMRI by censoring frames with framewise displacement (FD) > 0.5 mm and DVARS > 0.005^54^ and the number of remaining volumes did not differ between patients and controls (patients: mean 225.22 (SD 22.25), controls: mean 231.00 (SD 4.47), *p* = 0.405). No significant group and task interaction effect on motion parameters was found. The motion parameter characteristics are tabulated in Supplementary Table 2. We further controlled for mean absolute motion displacement in the FC analyses.

### Task-free and task-based functional connectivity derivation and analysis

For both task-free and task-based fMRI data, we parceled the brain into 144 regions of interest (ROIs)^55^ and extracted the BOLD time series of each ROI (averaged across all voxels in the ROI) for every participant. ROIs falling within a lesion or intersecting with the lesion were not excluded (see Supplementary Table 3 for the average frequency of lesion occurrence in the ipsilesional hemisphere). We then computed the Pearson’s correlation between the mean time series of every pair of ROIs. These correlation coefficients were Fisher’s r-to-z transformed to generate a 144 × 144 FC matrix for every participant for every condition (resting-state and four task conditions). FC of default, control, limbic, salience/ventral attention, dorsal attention, somatomotor, visual, temporoparietal, and subcortical networks were consequently derived by averaging the corresponding cells in the FC matrix. We also created a task-general FC matrix by concatenating the fMRI time-series data from all four task conditions. Therefore, every participant had six FC matrices representing task-free, task-general, AA, AU, PA, and PU conditions.

To investigate the group (patients vs controls) and task (rest vs task-general) interaction, we performed two-way repeated measure analysis of covariance with mean absolute motion displacement as a nuisance covariate using NBS toolbox with *p* < 0.05 FDR corrected. We chose PU motor task, the easiest condition among the four, as our reference condition and performed three separate two-way repeated measures ANCOVA to examine the effect of group and task (AA/AU/PA versus PU) as well as their interactions using NBS with *p* < 0.05 FDR corrected.

To compare group differences in FC strength at task-free and task-based conditions, we performed two-sample *t* tests on task-free and each of five task-based FC matrices between healthy controls and stroke patients using NBS, a nonparametric massive univariate method, controlling for mean absolute motion displacement, with a height threshold of *p* < 0.001 and a cluster-extent threshold of *p* < 0.05 (familywise-error corrected)^56^.

Four control analyses were performed. As the scan length differed between task-free and motor task-based fMRI, in control analysis I, we repeated the analysis by trimming each participant’s motor task fMRI images to maintain equal scan lengths as resting-state fMRI. In control analysis II, we matched the volumes of scans between task and resting-state fMRI given different TRs and repeated the analysis. As existing literature suggested global signal may contain information related to cognition and behavior^57^, we repeated our analysis based on the resting and task fMRI data without global signal regression in control analysis III. Lastly, as there were two left-handed stroke patients, we repeated the FC analyses controlling for handedness and the affected hand in control analysis IV.

### Task-related brain network reconfiguration analysis

Task-related brain network reconfiguration (i.e., spatial similarity between task-free and task-based FC matrices) was computed following a prior work^16^. Higher spatial similarity represents less task-related brain network reconfigurations compared to resting-state. Two-sample *t*-tests were used to compare the group difference in four network-specific task-related reconfigurations (i.e., AA, AU, PA, and PU) at the significance level of *p* < 0.05 (two-tailed) for every task.

### Association between baseline task-specific FC/reconfiguration and motor recovery

To investigate the association between baseline task-specific brain measures (i.e., FC and brain network reconfiguration) and motor recovery in chronic subcortical stroke patients, we built one stepwise multiple regression model for each task condition by including FC strengths or reconfiguration measures showing group differences as predictors (Figs. 2B and 3). Each intra- and inter-network FC measure (i.e., strength and reconfiguration) was further categorized into ipsilesional, contralesional, or inter-hemispheric. In terms of motor recovery, we calculated a normalized FMA change score representing motor gains after intervention: FMA_change_ = (FMA_post-intervention_ – FMA_pre-intervention_)/FMA_pre-intervention_. We performed stepwise multiple regression with normalized FMA change score as the dependent variable and FC or reconfiguration measures derived from networks showing group differences of each task (i.e., AA, AU, PA, and PU) as independent variables of interest. Age, sex, handedness, and time post-stroke were included as nuisance covariates. Therefore, there were a total of eight models for FC and reconfiguration of four tasks.

## Supplementary Information

Supplementary Information.

## Data Availability

The data that support the findings of this study are available from the corresponding author upon reasonable request.
