# Supplementary material for: Task-related brain functional network reconfigurations relate to motor recovery in chronic subcortical stroke
Source: Sci Rep. 2021 Apr 19;11:8442. doi: 10.1038/s41598-021-87789-5 (PMC8055891; doi:10.1038/s41598-021-87789-5)
Supplement: Supplementary file 1 — Supplementary Information. [file 41598_2021_87789_MOESM1_ESM.pdf]

Task-related brain functional network reconfigurations relate to motor recovery in chronic subcortical stroke: A pilot study

Hsiao-Ju Cheng, Kwun Kei Ng, Xing Qian, Fang Ji, Zhong Kang Lu, Wei Peng Teo, Xin Hong, Fatima Ali Nasrallah, Kai Keng Ang, Kai-Hsiang Chuang, Cuntai Guan, Haoyong Yu, Effie Chew†, and Juan Helen Zhou†

† joint senior authors

SUPPLEMENTARY MATERIALS

Supplementary Methods

Participants

Forty-two subjects were assessed for eligibility between June 2011 to January 2014. Eight of them declined to participate, five were excluded because they did not meet the inclusion criteria, and another ten were excluded because they did not meet the brain-computer interface (BCI) performance criteria. The remaining 19 subjects completed the training and magnetic resonance imaging (MRI) scans. Ten participants were allocated to the transcranial direct current stimulation (tDCS) group and nine were allocated to sham tDCS group.

Intervention

Stroke subjects were grouped according to their pre-training upper extremity Fugl-Meyer Assessment (FMA) scores (11–28 and 29–45) and then randomly assigned using a computer-generated random sequence into tDCS or sham group with matching FMA score between the groups. Each subject underwent ten 40-minute sessions of brain-computer interface-assisted motor imagery (MI-BCI) training over 2 weeks, each session preceded with either 20 minutes of tDCS or sham-tDCS (current ramped up and down to give subjects the sensation of the stimulation), applied at 1 mA through a pair of saline-soaked surface sponge electrodes (35 cm^2). The anode was placed over the ipsilesional primary motor cortex (M1) while the cathode over the contralesional M1, according to the initial exploration using TMS which identified the hotspot for activating the muscle of the hand and with reference to the International 10–20 Electrode Placement System for electroencephalography (EEG) electrode placement. The MI-BCI training involved mental imagery of a reaching task. Motor intention was detected using EEG, which triggered the movement of the stroke-affected arm using the Inmotion[2] MIT-Manus robot (Interactive Motion Technologies, MA, USA)30. As EEG signals were continuously recorded during the MI-BCI training and

tDCS may interfere the detection accuracy of EEG, the tDCS was applied prior to the MI-BCI training. The patient and the assessors were blinded to the tDCS condition.

Supplementary Results

Sample size estimation

To estimate the effect size and power based on our findings of multiple regression model built on AA to associate task-specific FC with motor recovery, we used G*Power 3.1.9.3 with settings of F tests, linear multiple regression: fixed model, R^2 deviation from zero, number of predictor: 2 (within ipliesional doral attention network, and between contralesional somatomotor and default networks). Our estimated effect size f^2 was 0.84 and power was 0.9. Based on this effect size and power, the estimated sample size for future study would be 19 stroke patients.

Supplementary Table 1. Stroke patient demographics and clinical information.

Intervention group	Subject ID	Age (years)	Sex	Handedness	Time post-stroke (months)	Lesioned hemisphere	Type of stroke
Real tDCS	1	29	Male	Left	12	Right	Ischemic
Real tDCS	5	54	Male	Right	28	Left	Ischemic
Real tDCS	6	38	Female	Right	29	Right	Hemorrhagic
Real tDCS	10	60	Female	Right	51	Right	Hemorrhagic
Real tDCS	15	48	Female	Right	49	Left	Hemorrhagic
Real tDCS	25	59	Male	Right	13	Left	Ischemic
Real tDCS	27	65	Male	Right	27	Left	Ischemic
Real tDCS	29	57	Female	Right	10	Left	Hemorrhagic
Real tDCS	35	47	Male	Right	10	Right	Ischemic
Real tDCS	37	65	Male	Right	86	Right	-
Sham tDCS	7	51	Male	Right	44	Right	Ischemic
Sham tDCS	9	39	Male	Right	25	Left	Ischemic
Sham tDCS	11	59	Male	Right	52	Right	Hemorrhagic
Sham tDCS	18	70	Female	Right	19	Right	Ischemic
Sham tDCS	19	59	Male	Right	44	Right	Ischemic
Sham tDCS	21	58	Male	Left	29	Left	Ischemic
Sham tDCS	30	58	Male	Right	25	Right	Hemorrhagic
Sham tDCS	31	47	Male	Right	10	Left	Ischemic
Sham tDCS	32	67	Male	Right	52	Right	Ischemic

Note. The type of stroke of subject 37 was missing.

Supplementary Table 2. Motion parameter characteristics by group.

	**Healthy Controls (n = 11)**	**Stroke Patients (n = 18)**	***p*-value (2-tailed)**
Mean absolute motion displacement of task-free fMRI after motion scrubbing (mm), mean (SD)	0.36 (0.23)	0.22 (0.15)	0.053
Mean relative motion displacement of task-free fMRI after motion scrubbing (mm), mean (SD)	0.08 (0.03)	0.08 (0.04)	0.904
Mean absolute motion displacement of active-affected task (mm), mean (SD)	0.31 (0.17)	0.48 (0.41)	0.203
Mean relative motion displacement of active-affected task (mm), mean (SD)	0.09 (0.02)	0.13 (0.07)	0.045*
Mean absolute motion displacement of active-unaffected task (mm), mean (SD)	0.32 (0.13)	0.31 (0.18)	0.910
Mean relative motion displacement of active-unaffected task (mm), mean (SD)	0.09 (0.02)	0.11 (0.07)	0.416
Mean absolute motion displacement of passive-affected task (mm), mean (SD)	0.21 (0.12)	0.20 (0.16)	0.837
Mean relative motion displacement of passive-affected task (mm), mean (SD)	0.07 (0.03)	0.09 (0.08)	0.636
Mean absolute motion displacement of passive-unaffected task (mm), mean (SD)	0.23 (0.06)	0.17 (0.08)	0.036*
Mean relative motion displacement of passive-unaffected task (mm), mean (SD)	0.07 (0.02)	0.07 (0.03)	0.974

Notes. No group and task interaction effect was revealed by two-way repeated measure analysis of variance. '*' represents $p < 0.05$ revealed by two-sample t-tests.

Supplementary Table 3. The average frequency of lesion occurrence in the ipsilesional hemisphere.

Network	Brain regions based on Yeo et al. (2011)	Frequency of lesion occurrence (%), mean (SD)
Default	Default A: inferior parietal lobule	11.35 ± 24.12
	Default A: dorsal prefrontal cortex	1.85 ± 4.10
	Default A: precuneus posterior cingulate cortex	0.93 ± 1.66
	Default A: medial prefrontal cortex	0.29 ± 0.91
	Default B: temporal	10.52 ± 21.31
	Default B: inferior parietal lobule	15.17 ± 29.20
	Default B: dorsal prefrontal cortex	0.49 ± 1.61
	Default B: lateral prefrontal cortex	6.26 ± 23.45
	Default B: ventral prefrontal cortex	6.21 ± 22.56
	Default C: inferior parietal lobule	10.83 ± 22.42
	Default C: retrosplenial	1.83 ± 3.68
	Default C: parahippocampal cortex	2.85 ± 7.18
Control	Control A: temporal	2.06 ± 7.29
	Control A: intraparietal sulcus	12.11 ± 24.50
	Control A: dorsal prefrontal cortex	8.78 ± 23.65
	Control A: lateral prefrontal cortex	14.22 ± 25.28
	Control A: lateral ventral prefrontal cortex	8.82 ± 23.18
	Control A: mid-cingulate	4.55 ± 0.00
	Control B: temporal	6.59 ± 15.49
	Control B: inferior parietal lobule	7.63 ± 22.39
	Control B: dorsal prefrontal cortex	5.68 ± 14.25
	Control B: lateral prefrontal cortex	5.64 ± 22.27
	Control B: lateral ventral prefrontal cortex	1.65 ± 3.37
	Control B: medial posterior prefrontal cortex	1.72 ± 0.00
	Control C: precuneus	0.36 ± 0.79
	Control C: cingulate posterior	1.01 ± 0.00
Salience/Ventral attention	Salience/ventral attention A: parietal operculum	13.45 ± 26.16
	Salience/ventral attention A: frontal operculum	12.29 ± 25.29
	Salience/ventral attention A: insula	32.84 ± 40.93
	Salience/ventral attention A: parietal medial	11.43 ± 11.45
	Salience/ventral attention A: frontal medial	3.52 ± 10.65
	Salience/ventral attention B: inferior parietal lobule	11.24 ± 26.78
	Salience/ventral attention B: dorsal prefrontal cortex	10.07 ± 16.20
	Salience/ventral attention B: lateral prefrontal cortex	2.68 ± 7.27
	Salience/ventral attention B: insula	17.79 ± 31.51
	Salience/ventral attention B: orbital frontal cortex	12.87 ± 20.11
	Salience/ventral attention B: medial posterior prefrontal cortex	1.61 ± 2.90
Dorsal attention	Dorsal attention A: temporal occipital	3.48 ± 5.96
	Dorsal attention A: parietal occipital	9.06 ± 22.28
	Dorsal attention A: superior parietal lobe	3.01 ± 6.75
	Dorsal attention B: temporal occipital	8.17 ± 23.23

	Dorsal attention B: posterior central	8.08 ± 17.21
	Dorsal attention B: frontal eye field	16.15 ± 29.37
	Dorsal attention B: precentral ventral	11.40 ± 24.91
Somatomotor	Somatomotor A	6.07 ± 20.53
	Somatomotor B: central	15.16 ± 27.54
	Somatomotor B: S2	18.91 ± 25.79
	Somatomotor B: insula	58.44 ± 41.76
	Somatomotor B: auditory	20.54 ± 33.09
Subcortical	AAL_37_Hippocampus	13.92 ± 20.92
	AAL_41_Amygdala	18.82 ± 28.29
	striatum_2	63.44 ± 39.40
	striatum_4	46.95 ± 33.38
	striatum_5	9.88 ± 21.11
	striatum_6	35.85 ± 28.26
	striatum_7	24.91 ± 25.67
	thalamus_1	19.43 ± 26.60
	thalamus_2	25.40 ± 23.48
	thalamus_3	27.45 ± 29.50
	thalamus_4	29.70 ± 22.08
	thalamus_5	9.51 ± 13.12
	thalamus_6	17.99 ± 25.60
	thalamus_7	6.88 ± 14.21
Visual	Central Visual: striate cortex	2.17 ± 5.77
	Central Visual: extrastriate	3.76 ± 7.99
	Peripheral Visual: striate	6.82 ± 16.44
	Peripheral Visual: inferior extrastriate cortex	2.21 ± 4.35
	Peripheral Visual: superior extrastriate cortex	2.84 ± 8.58
Limbic	LimbicA: temporal pole	5.64 ± 17.92
	LimbicB: orbital frontal cortex	0.91 ± 2.37
Temporoparietal	Temporoparietal	14.78 ± 30.68

Note. Frequency of lesion occurrence (%) was calculated by dividing the number of voxels falling within the lesion mask by total number of voxels within the region of interest for every region of interest.

Supplementary Table 4. Group difference in reconfiguration analyses at whole brain and network level by task.

	Task	Group	Correlation Coefficient Mean ± SD	*p*-value (2-tailed)
**Whole-Brain**	Active-Affected	Healthy controls	0.39 ± 0.07	0.150
		Stroke patients	0.35 ± 0.08	
	Active-Unaffected	Healthy controls	0.40 ± 0.07	0.124
		Stroke patients	0.36 ± 0.07	
	Passive-Affected	Healthy controls	0.44 ± 0.07	0.131
		Stroke patients	0.39 ± 0.09	
	Passive-Unaffected	Healthy controls	0.44 ± 0.05	0.077
		Stroke patients	0.38 ± 0.08	
**Default-default**	Active-Affected	Healthy controls	0.44 ± 0.11	0.304
		Stroke patients	0.39 ± 0.12	
	Active-Unaffected	Healthy controls	0.44 ± 0.10	0.997
		Stroke patients	0.44 ± 0.12	
	Passive-Affected	Healthy controls	0.47 ± 0.10	0.575
		Stroke patients	0.44 ± 0.15	
	Passive-Unaffected	Healthy controls	0.47 ± 0.10	0.149
		Stroke patients	0.41 ± 0.11	
**Control-control**	Active-Affected	Healthy controls	0.48 ± 0.06	0.198
		Stroke patients	0.42 ± 0.14	
	Active-Unaffected	Healthy controls	0.49 ± 0.07	0.487
		Stroke patients	0.46 ± 0.15	
	Passive-Affected	Healthy controls	0.50 ± 0.06	0.582
		Stroke patients	0.47 ± 0.14	
	Passive-Unaffected	Healthy controls	0.51 ± 0.06	0.224
		Stroke patients	0.46 ± 0.11	
**SalVenAttn-SalVenAttn**	Active-Affected	Healthy controls	0.44 ± 0.09	0.420
		Stroke patients	0.40 ± 0.14	
	Active-Unaffected	Healthy controls	0.46 ± 0.09	0.117
		Stroke patients	0.39 ± 0.11	
	Passive-Affected	Healthy controls	0.47 ± 0.09	0.203
		Stroke patients	0.40 ± 0.15	
	Passive-Unaffected	Healthy controls	0.47 ± 0.10	0.340
		Stroke patients	0.43 ± 0.15	
**DorsAttn-DorsAttn**	Active-Affected	Healthy controls	0.34 ± 0.14	0.093
		Stroke patients	0.43 ± 0.15	
	Active-Unaffected	Healthy controls	0.36 ± 0.14	0.295
		Stroke patients	0.43 ± 0.17	
	Passive-Affected	Healthy controls	0.46 ± 0.18	0.724
		Stroke patients	0.49 ± 0.17	
	Passive-Unaffected	Healthy controls	0.43 ± 0.17	0.300
		Stroke patients	0.49 ± 0.13	
**SomMot-SomMot**	Active-Affected	Healthy controls	0.64 ± 0.13	0.557
		Stroke patients	0.60 ± 0.17	
	Active-Unaffected	Healthy controls	0.63 ± 0.11	0.317

		Stroke patients	0.57 ± 0.18	
	Passive-Affected	Healthy controls	0.67 ± 0.16	0.235
		Stroke patients	0.57 ± 0.25	
	Passive-Unaffected	Healthy controls	0.66 ± 0.13	0.132
		Stroke patients	0.55 ± 0.22	
**Subcortical-subcortical**	Active-Affected	Healthy controls	0.59 ± 0.11	0.004*
		Stroke patients	0.47 ± 0.09	
	Active-Unaffected	Healthy controls	0.57 ± 0.11	0.068
		Stroke patients	0.50 ± 0.10	
	Passive-Affected	Healthy controls	0.60 ± 0.13	0.012*
		Stroke patients	0.49 ± 0.08	
	Passive-Unaffected	Healthy controls	0.62 ± 0.09	0.004*
		Stroke patients	0.50 ± 0.10	
**Default-control**	Active-Affected	Healthy controls	0.42 ± 0.10	0.130
		Stroke patients	0.35 ± 0.12	
	Active-Unaffected	Healthy controls	0.43 ± 0.11	0.316
		Stroke patients	0.39 ± 0.11	
	Passive-Affected	Healthy controls	0.44 ± 0.08	0.232
		Stroke patients	0.39 ± 0.10	
	Passive-Unaffected	Healthy controls	0.44 ± 0.09	0.058
		Stroke patients	0.38 ± 0.09	
**Default-SomMot**	Active-Affected	Healthy controls	0.26 ± 0.13	0.094
		Stroke patients	0.16 ± 0.17	
	Active-Unaffected	Healthy controls	0.29 ± 0.09	0.019*
		Stroke patients	0.20 ± 0.11	
	Passive-Affected	Healthy controls	0.27 ± 0.17	0.503
		Stroke patients	0.22 ± 0.18	
	Passive-Unaffected	Healthy controls	0.31 ± 0.11	0.037*
		Stroke patients	0.18 ± 0.17	
**Default-SalAttn**	Active-Affected	Healthy controls	0.27 ± 0.08	0.442
		Stroke patients	0.24 ± 0.11	
	Active-Unaffected	Healthy controls	0.28 ± 0.10	0.762
		Stroke patients	0.27 ± 0.09	
	Passive-Affected	Healthy controls	0.32 ± 0.09	0.662
		Stroke patients	0.30 ± 0.14	
	Passive-Unaffected	Healthy controls	0.34 ± 0.08	0.122
		Stroke patients	0.28 ± 0.11	
**Control-subcortical**	Active-Affected	Healthy controls	0.19 ± 0.17	0.333
		Stroke patients	0.13 ± 0.16	
	Active-Unaffected	Healthy controls	0.20 ± 0.15	0.254
		Stroke patients	0.13 ± 0.16	
	Passive-Affected	Healthy controls	0.21 ± 0.10	0.371
		Stroke patients	0.17 ± 0.12	
	Passive-Unaffected	Healthy controls	0.25 ± 0.13	0.026*
		Stroke patients	0.14 ± 0.13	
**Control-DorsAttn**	Active-Affected	Healthy controls	0.42 ± 0.08	0.896
		Stroke patients	0.42 ± 0.11	
	Active-Unaffected	Healthy controls	0.39 ± 0.09	0.373

		Stroke patients	0.43 ± 0.11	
	Passive-Affected	Healthy controls	0.45 ± 0.09	0.894
		Stroke patients	0.45 ± 0.12	
	Passive-Unaffected	Healthy controls	0.42 ± 0.09	0.489
		Stroke patients	0.44 ± 0.11	
**SalVenAttn-subcortical**	Active-Affected	Healthy controls	0.28 ± 0.11	0.015*
		Stroke patients	0.16 ± 0.12	
	Active-Unaffected	Healthy controls	0.25 ± 0.16	0.088
		Stroke patients	0.15 ± 0.15	
	Passive-Affected	Healthy controls	0.27 ± 0.09	0.024*
		Stroke patients	0.16 ± 0.13	
	Passive-Unaffected	Healthy controls	0.25 ± 0.10	0.037*
		Stroke patients	0.16 ± 0.10	
**SalAttn-SomMot**	Active-Affected	Healthy controls	0.37 ± 0.13	0.543
		Stroke patients	0.40 ± 0.16	
	Active-Unaffected	Healthy controls	0.38 ± 0.13	0.902
		Stroke patients	0.39 ± 0.11	
	Passive-Affected	Healthy controls	0.44 ± 0.14	0.691
		Stroke patients	0.42 ± 0.12	
	Passive-Unaffected	Healthy controls	0.41 ± 0.14	0.895
		Stroke patients	0.42 ± 0.15	
**SomMot-subcortical**	Active-Affected	Healthy controls	0.28 ± 0.18	0.046*
		Stroke patients	0.14 ± 0.17	
	Active-Unaffected	Healthy controls	0.32 ± 0.19	0.005*
		Stroke patients	0.09 ± 0.20	
	Passive-Affected	Healthy controls	0.36 ± 0.23	0.027*
		Stroke patients	0.17 ± 0.19	
	Passive-Unaffected	Healthy controls	0.37 ± 0.23	0.016*
		Stroke patients	0.16 ± 0.20	

Notes. '*' represents $p < 0.05$. Abbreviations: DorsAttn = dorsal attention; SalAttn = salience/ventral attention + dorsal attention; SalVenAttn = salience/ventral attention; SomMot = somatomotor.

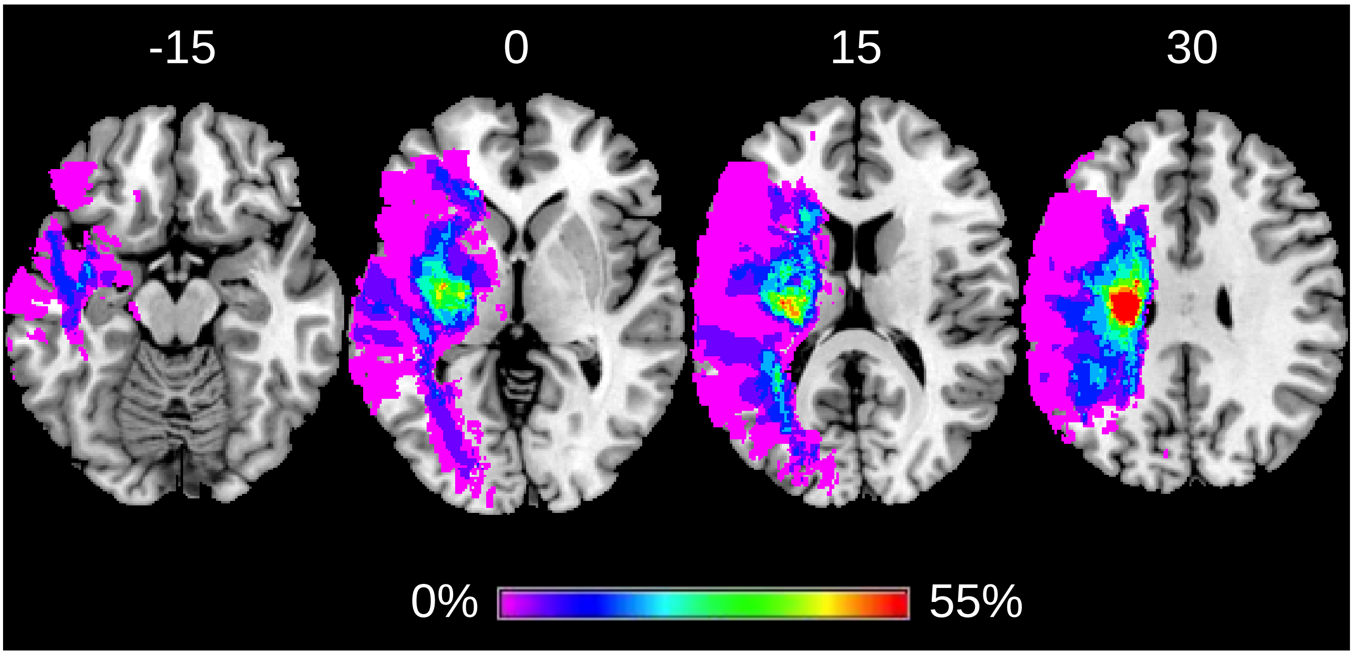

Supplementary Figure 1. The lesion overlapping map in chronic subcortical stroke patients. Firstly, lesions in the right hemisphere were flipped to the left hemisphere. All binarized lesion maps were then registered and overlaid to the Montreal Neurological Institute (MNI) 152 template. Frequency of occurrence was denoted in the color bar. Core lesions were predominantly located in subcortical regions and the penumbra extended to cortical cortex.

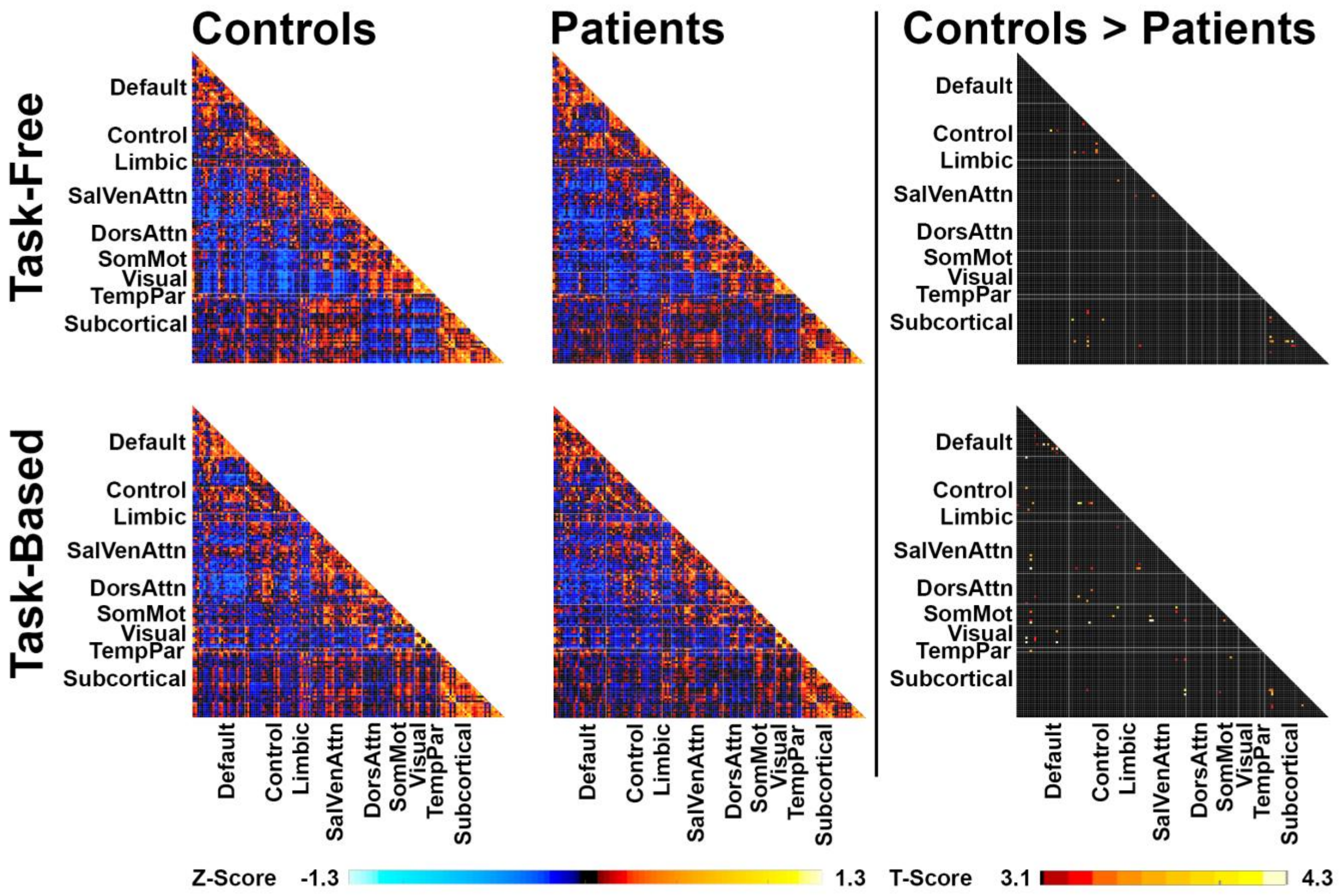

Supplementary Figure 2. More widespread network disruptions in task-based condition than task-free condition in stroke patients demonstrated by functional connectivity strength.

Compared to age-matched healthy controls, stroke patients showed lower intra-network FC in control, SalVenAttn, and subcortical networks as well as lower inter-network FC between control and subcortical networks during the task-free condition. During the task-general condition, stroke patients had more widespread network disruptions than resting-state, including lower intra-network FC in default, control, SalVenAttn, SomMot, and subcortical networks. Additionally, lower inter-network FC was found between default and other networks (except limbic and subcortical), control and other networks (except limbic, visual, and TempPar), as well as SalVenAttn and SomMot and subcortical networks.

Abbreviations: DorsAttn = dorsal attention; FC = functional connectivity; SalVenAttn = salience/ventral attention; SomMot = somatomotor; TempPar = temporoparietal.

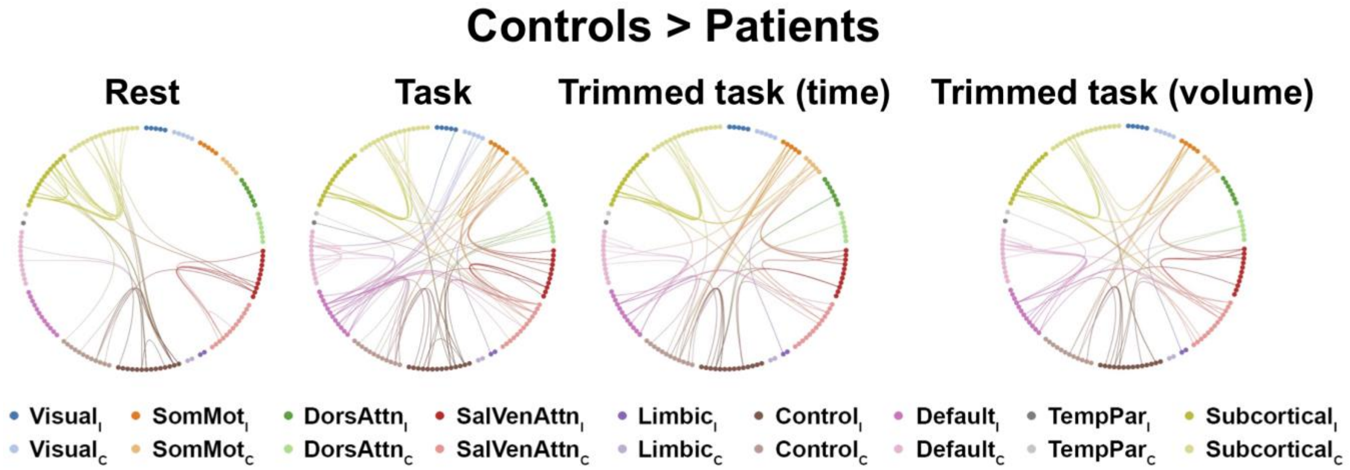

Supplementary Figure 3. More widespread network disruptions in task-based condition than task-free condition in stroke patients was independent of scan length.

Abbreviations: C = contralesional; DorsAttn = dorsal attention; I = ipsilesional; SalVenAttn = salience/ventral attention; SomMot = somatomotor; TempPar = temporoparietal.

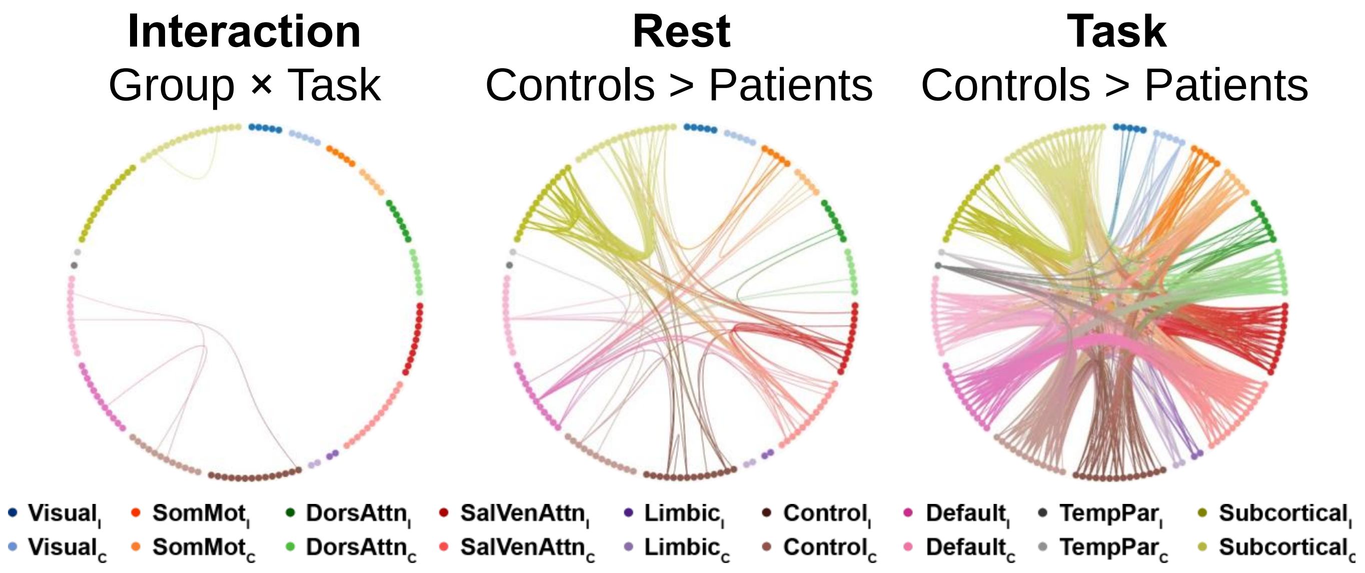

Supplementary Figure 4. More widespread network disruptions in task-based condition than task-free condition in stroke patients independent of global signal regression.

(Left panel) Significant interaction effects of group (patients vs controls) and task (rest vs task) were found in intra-network FC within subcortical network and in inter-network FC between default and control networks. (Middle and Right panel) Compared to age-matched healthy controls, stroke patients had widespread network disruptions at rest condition, including lower intra-network FC in default, control, SalVenAttn, and subcortical networks. Additionally, lower inter-network FC was found between ipsilesional default and contralesional SalVenAttn and bilateral SomMot networks, ipsilesional control and bilateral subcortical networks, contralesional SalVenAttn and bilateral subcortical networks, as well as ipsileional SalVenAttn and bilateral subcortical networks. In comparison, stroke patients showed extensive lower FC across all inter- and intra-networks during task. These findings are similar to those found using global signal-regressed data, supporting that stroke patients indeed had more widespread network disruptions in task-based condition than task-free condition.

Abbreviations: AA = active-affected; AU = active-unaffected; C = contralesional; DorsAttn = dorsal attention; FC = functional connectivity; I = ipsilesional; PA = passive-affected; PU = passive-unaffected; SalVenAttn = salience/ventral attention; SomMot = somatomotor; TempPar = temporoparietal.

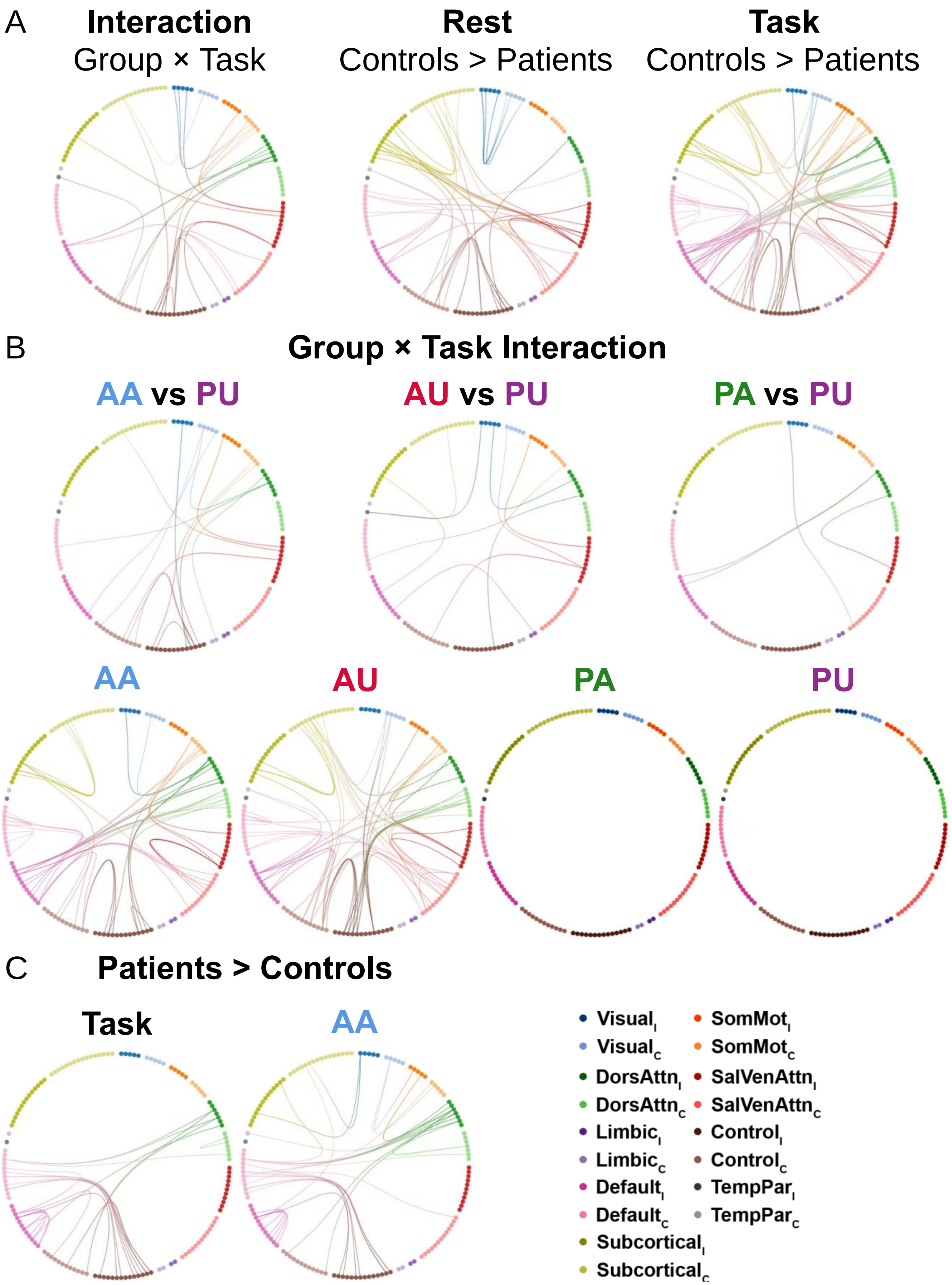
A
Interaction
Group × Task
Rest
Controls > Patients
Task
Controls > Patients
B
Group × Task Interaction
AA vs PU
AU vs PU
PA vs PU
AA
AU
PA
PU
C
Patients > Controls
Task
AA
Visual$_I$
Visual$_C$
DorsAttn$_I$
DorsAttn$_C$
Limbic$_I$
Limbic$_C$
Default$_I$
Default$_C$
Subcortical$_I$
Subcortical$_C$
SomMot$_I$
SomMot$_C$
SalVenAttn$_I$
SalVenAttn$_C$
Control$_I$
Control$_C$
TempPar$_I$
TempPar$_C$

Supplementary Figure 5. Disrupted functional connectivity at different task demands in stroke patients after controlling for handedness and the affected hand. (A) (Left panel) Significant interaction effects of group (patients vs controls) and task (rest vs task) were found (height threshold uncorrected $p < 0.01$). (Middle and Right panel) Compared to healthy controls, stroke patients showed more widespread network disruptions in task-based condition than task-free condition. (B) (Upper panel) Significant interaction effects of group (patients vs controls) and task (AA/AU/PA vs PU) were found (height threshold uncorrected $p < 0.01$). (Lower panel) Compared to healthy controls, stroke patients showed widespread network disruptions in AA and AU conditions. (C) Stroke patients demonstrated anti-correlation pattern in task and AA.

Abbreviations: AA = active-affected; AU = active-unaffected; C = contralesional; DorsAttn = dorsal attention; FC = functional connectivity; I = ipsilesional; PA = passive-affected; PU = passive-unaffected; SalVenAttn = salience/ventral attention; SomMot = somatomotor; TempPar = Temporoparietal.

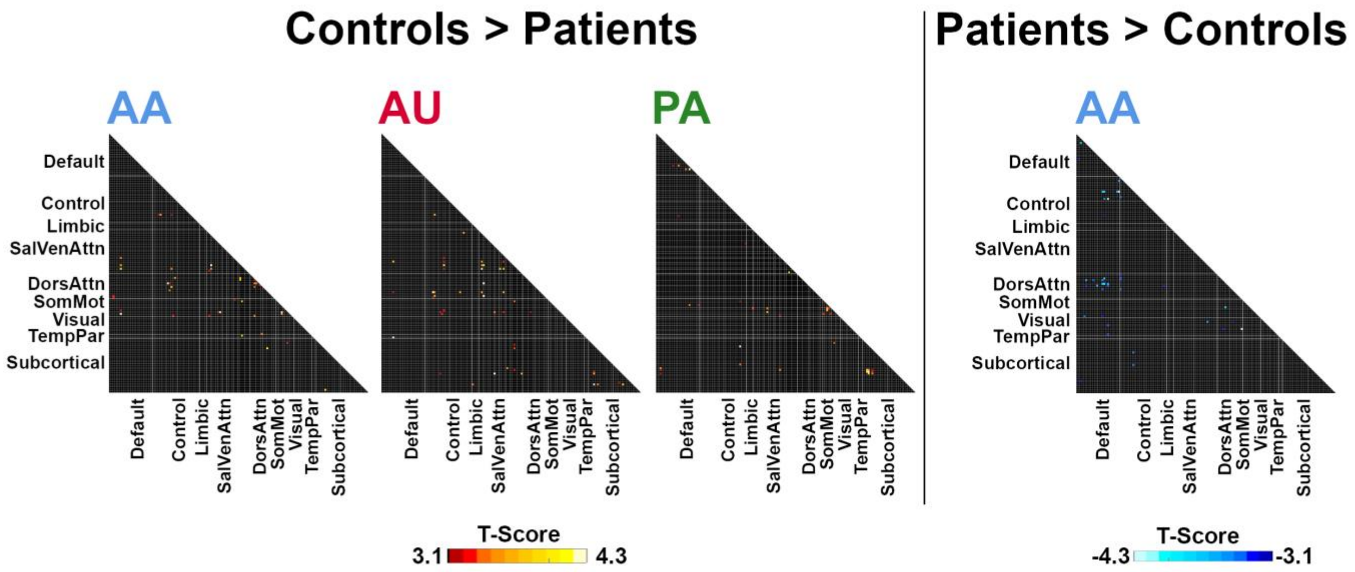

Supplementary Figure 6. Disrupted intra- and inter-network functional connectivity at different task demands in stroke patients demonstrated by functional connectivity strength.

Compared to age-matched healthy controls, stroke patients showed more disrupted intra- and inter-network FC during AA, AU, and PA tasks. During AA, stroke patients demonstrated lower FC in ipsilesional default as well as bilateral control, SalVenAttn, DorsAttn, SomMot, and subcortical networks. Remarkably, stroke patients presented additionally lower DorsAttn intra-network FC in AA compared to other tasks. Along with the decremental task demands, stroke patients showed less intra- and inter-network FC disruption. Especially for AA task, stroke patients showed more anti-correlation than healthy controls in default, control, and DorsAttn network.

Abbreviations: DorsAttn = dorsal attention; FC = functional connectivity; SalVenAttn = salience/ventral attention; SomMot = somatomotor; TempPar = temporoparietal.

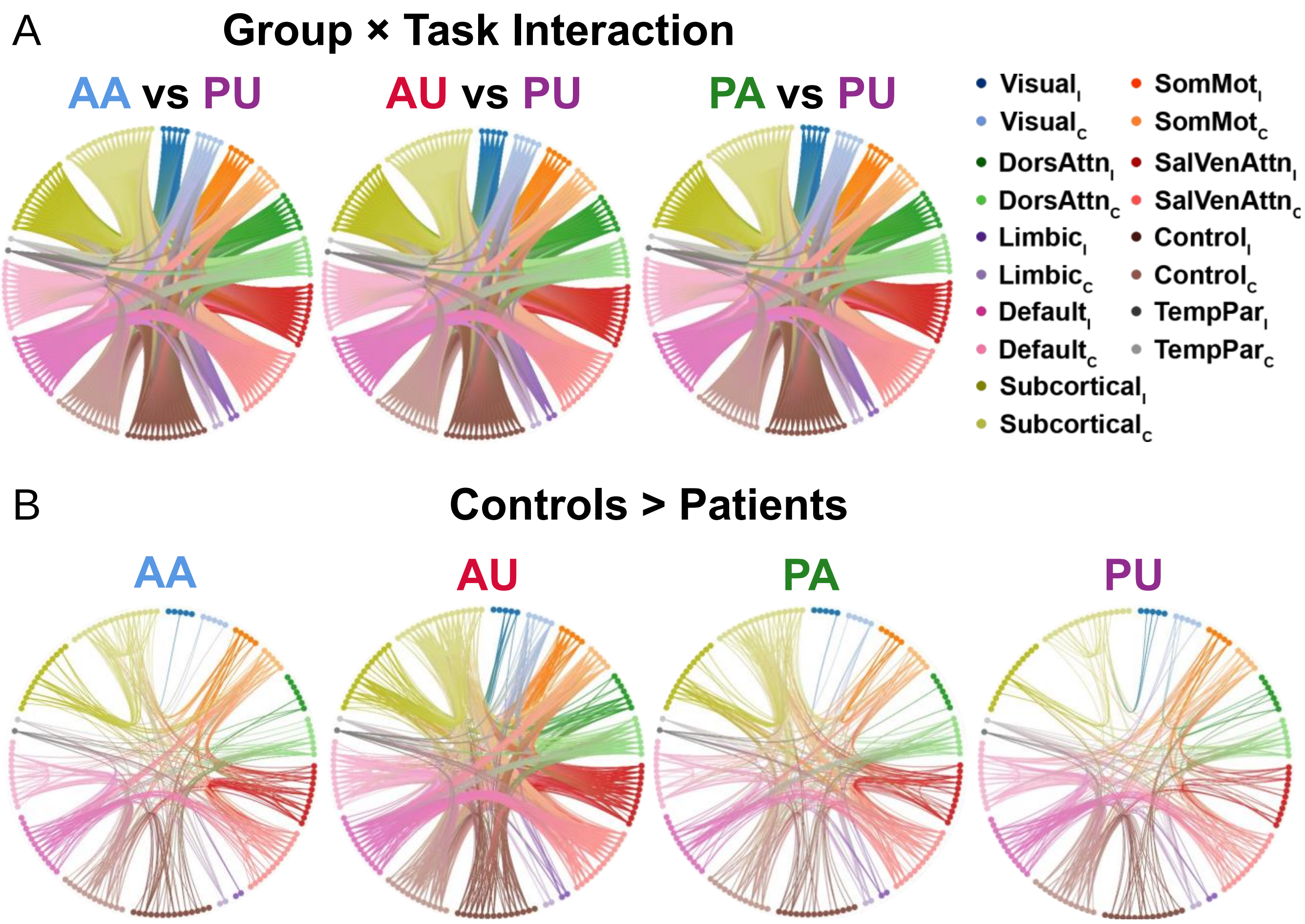

Supplementary Figure 7. Disrupted intra- and inter-network functional connectivity at different task demands in stroke patients independent of global signal regression.

(A) Significant group and task interaction effects were found between AA/AU/PA and PU. (B) Compared to age-matched healthy controls, stroke patients had widespread network disruptions at all four task conditions. Using data without global signal regression, we still demonstrated different patterns of network disruptions in chronic subcortical stroke patients.

Abbreviations: AA = active-affected; AU = active-unaffected; C = contralesional; DorsAttn = dorsal attention; FC = functional connectivity; I = ipsilesional; PA = passive-affected; PU = passive-unaffected; SalVenAttn = salience/ventral attention; SomMot = somatomotor; TempPar = temporoparietal.

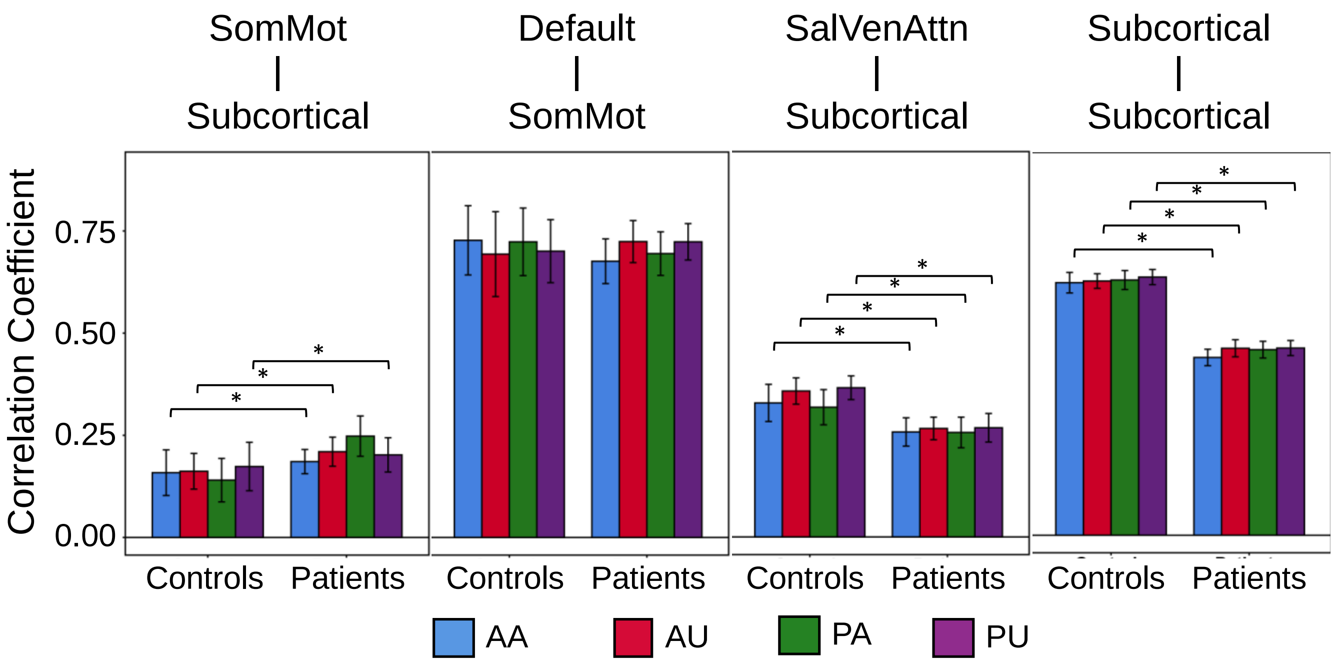

Supplementary Figure 8. Altered network-specific reconfiguration at different task demands in stroke patients without regressing out global signal. At the network-level, stroke patients presented network-specific brain network reconfiguration compared to healthy controls. Stroke patients had higher task-related reconfiguration (i.e., lower correlation coefficient) between SomMot and subcortical networks across all tasks except PA. Stroke patients also showed higher reconfiguration between SalVenAttn and subcortical inter-network as well as subcortical intra-network across all tasks. Data are presented as mean ± standard error, *$p < 0.05$. Abbreviations: AA = active-affected; AU = active-unaffected; PA = passive-affected; PU = passive-unaffected; SalVenAttn = salience/ventral attention.

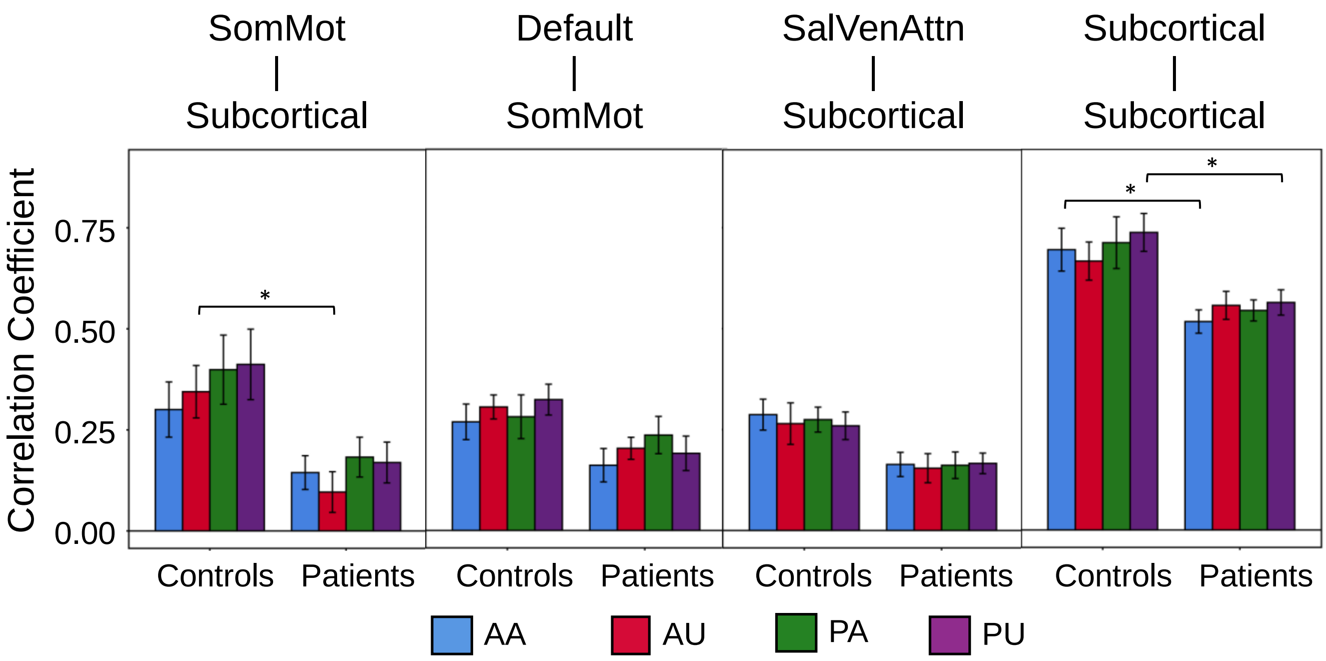

Supplementary Figure 9. Altered network-specific reconfiguration at different task demands in stroke patients after controlling for handedness and the affected hand. At the network-level, stroke patients presented network-specific brain network reconfiguration compared to healthy controls. Stroke patients had higher task-related reconfiguration (i.e., lower correlation coefficient) between SomMot and subcortical networks during AU. Stroke patients also showed higher reconfiguration within subcortical network during AA and PU tasks. Data are presented as mean ± standard error, *$p < 0.05$.

Abbreviations: AA = active-affected; AU = active-unaffected; PA = passive-affected; PU = passive-unaffected; SalVenAttn = salience/ventral attention.

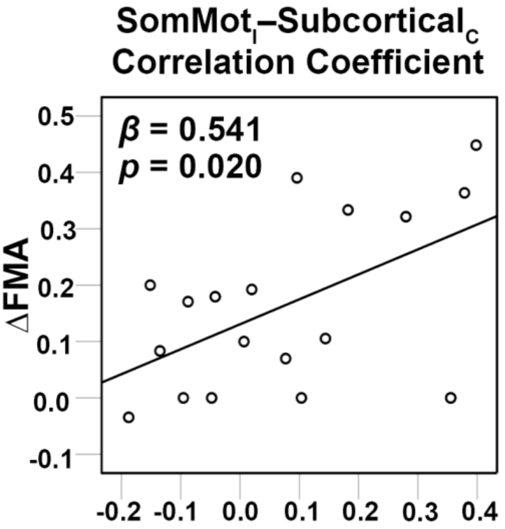

Supplementary Figure 10. Network-specific reconfiguration was associated with motor recovery in stroke patients.

More efficient task-related brain network reconfiguration (i.e. higher correlation coefficient) between ipsilesional SomMot and contralesional subcortical network was associated with more motor recovery. Abbreviation: C = contralesional; FMA = Fugl-Meyer Assessment; I = ipsilesional; SomMot = somatomtor.
